# Ab-SELDON: Leveraging Diversity Data for an Efficient Automated Computational Pipeline for Antibody Design

**DOI:** 10.1021/acs.jcim.5c01924

**Published:** 2026-01-20

**Authors:** Jean V. Sampaio, Andrielly H. S. Costa, Aline O. Albuquerque, Júlia S. Souza, Diego S. Almeida, Eduardo M. Gaieta, Matheus V. Almeida, Geraldo R. Sartori, João H. M. Silva

**Affiliations:** † Laboratory of Structural and Functional Biology Applied to Biopharmaceuticals, 611310 Fundação Oswaldo Cruz, Fiocruz Ceará, Eusébio 61773-270, Brazil; ‡ Instituto Oswaldo Cruz, Fiocruz, Rio de Janeiro, Rio de Janeiro 21040-900, Brazil; § Federal University of Ceará, Fortaleza, Ceará 60020-181, Brazil; ∥ São Carlos Institute of Physics, 117186 University of São Paulo, São Carlos, São Paulo 13566-590, Brazil; ⊥ Pasteur-Fiocruz Center on Immunology and Immunotherapy, Fundação Oswaldo Cruz, Fiocruz Ceará, Eusébio 61773-270, Brazil

## Abstract

The utilization of predictive tools has become increasingly prevalent in the development of biopharmaceuticals, reducing the time and cost of research. However, most methods for computational antibody design are hampered by their reliance on scarcely available antibody structures, potential for immunogenic modifications, and a restricted exploration of the paratope’s potential chemical and conformational space. We propose Ab-SELDON, a modular and easily customizable antibody design pipeline capable of iteratively optimizing an antibody–antigen (Ab–Ag) interaction in five different modification steps, including CDR and framework grafting, and mutagenesis. The optimization process is guided by diversity data collected from millions of publicly available human antibody sequences. This approach enhanced the exploration of the chemical and conformational space of the paratope during computational tests involving the optimization of an anti-HER2 antibody. Optimization of another antibody against Gal-3BP stabilized the Ab-Ag interaction in molecular dynamics simulations at lower runtime than alternative pipelines. Tests with SKEMPI’s Ab-Ag mutations also demonstrated the pipeline’s ability to correctly identify the effect of the majority of mutations, especially multipoint and those that increased binding affinity. This freely available pipeline presents a new approach for computationally efficient and automated *in silico* antibody design, thereby facilitating the development of new biopharmaceuticals.

## Graphical abstract

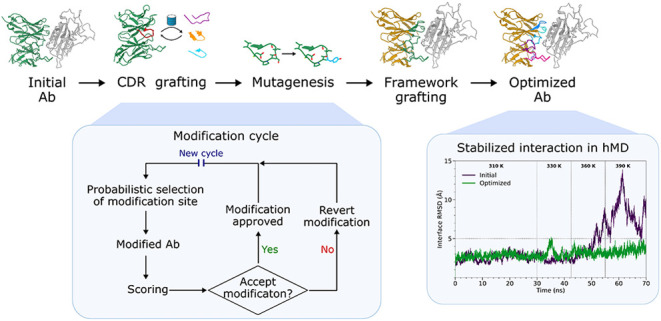

## 1 Introduction

Monoclonal antibodies are widely used to treat cancers, infectious diseases, and autoimmune disorders. They have become the leading class of biopharmaceuticals, with nearly 200 therapeutic antibodies currently approved or under regulatory review.^1^ This has led to the development of computational tools that can help reduce both the time and cost of rational antibody design compared with experimental approaches.^2,3^

Classical computational antibody design methods often combine CDR grafting and point mutations, followed by their evaluation. CDR grafting allows extensive exploration of the chemical and conformational space of CDRs with relatively few tests, analogous to the biological V­(D)­J recombination. Subsequently, point mutations can be introduced to further optimize the antibody–antigen (Ab-Ag) interaction, similarly to somatic hypermutation (SHM).^3,4^

A popular method among these is Rosetta Antibody Design (RAbD), which has been successfully used to generate anti-SARS-CoV-2 antibodies.^5^ RAbD grafts CDR structures from canonical clusters in the PyIgClassify database onto an antibody framework followed by mutagenesis. It operates on a series of modification cycles, where the changes are evaluated using an energy function and the Metropolis Monte Carlo criterion.^4^ Another method, OptMAVEn 2.0, assembles the antibody variable region from structural parts representing the V, D, and J regions, mimicking V­(D)­J recombination.

Both RAbD and OptMAVEn rely on a relatively small number of experimentally determined antibody structures as their starting points, restricting their exploration of the paratope chemical and conformational space. In the case of OptMAVEn, this is especially relevant to CDRs 1 and 2 because they are part of the V region and, therefore, are always grafted together.^4,6^

Rangel et al. addressed this limitation by grafting CDR-like fragments from various Protein Data Bank (PDB) proteins, including nonhuman and nonimmunoglobulin, broadening modification possibilities. Despite high antigen affinity, the authors noted that sequences from nonhuman proteins may lead to immunogenic antibodies, limiting their therapeutic potential.^7^ More recently, Barletta et al. published a modular platform for high-throughput parallel generation of multiple antibody mutation lineages, called Locuaz. However, this algorithm is limited to point mutations and lacks a fragment or CDR grafting step, restricting the exploration of different CDR lengths or conformations.^8^

A novel class of deep learning (DL) algorithms tailored for antibody design has recently emerged. In particular, some diffusion-based methods like EAGLE, DiffAb and RFantibody show promise among DL methods that account for the epitope during sequence-structure codesign. However, current DL-based approaches have low success rates, which can be less than 1% when designing antibodies with improved affinity against several targets, especially those without a known initial binder, with few considering the target epitope during design.^2,9–12^

Part of the reason for these low success rates lies in the inherent difficulty of predicting Ab–Ag complex structures using deep learning (DL) models. These models depend on the limited number of experimentally resolved Ab–Ag structures currently available for training. In recent years, algorithms such as AlphaFold 3 (AF3),^13^ Boltz-1,^14^ and Chai-1 ^15^ have achieved notable improvements over AlphaFold 2 in modeling Ab–Ag complexes. However, their performance remains constrained, with even the best-performing method, AlphaFold 3, producing acceptable or higher-quality models in fewer than 35% of tested cases in a recent study.^16^

To overcome the limitations of existing antibody design tools, we developed Ab-SELDON (Antibody Structural Enhancement Leveraging Diversity for Optimization of iNteractions), a computationally efficient and easily customizable Python-based modular pipeline for antibody design and optimization. This easy-to-use pipeline works in iterative cycles. In each cycle, modifications to the antibody sequence are introduced, the new sequence is modeled, followed by a minimization of the resulting Ab-Ag complex structure. The evaluation of the proposed modification uses either the CSM-AB or the REF15 scoring functions, combined with either the Metropolis criterion or two other possible modes for approval.^17^

In its default mode of operation, Ab-SELDON begins with a rough optimization of the starting Ab-Ag interaction through three different CDR grafting steps, using naïve antibody sequences taken from the Observed Antibody Space (OAS) database.^18^ It then fine-tunes the interaction through a mutagenesis step and a final framework swap step, both based on data from memory antibody sequences in OAS. Olsen et al. have previously demonstrated the importance of utilizing memory antibody data for optimization tasks in order to avoid germline bias and emphasize mutations that improve affinity and specificity toward desired antigens.^19^

Users can select the steps they wish to execute and the order in which they are performed. To improve the exploration of the antibody conformational space and optimization efficiency, Ab-SELDON probabilistically selects, in each cycle, a CDR for modification based on the relative diversity of the six CDRs calculated from OAS sequences.

This optimization process allows for extensive exploration of the chemical and conformational space of the antibody variable region, including the framework, resulting in a comprehensive and easily customizable antibody design pipeline.

## 2 Methods

All third-party software tools were executed with default parameters, unless explicitly specified otherwise. A table summarizing the nomenclature of the different modules/steps and data sets used by the pipeline can be found on the Supporting Information (Table S1).

### 2.1 Antibody Diversity Analysis and Sequence Data Sets

#### 2.1.1 Collecting and Filtering Sequences

To optimize the antibody modification process in Ab-SELDON, we collected diversity data from millions of experimentally determined unpaired antibody sequences in the Observed Antibody Space database,^18^ excluding sequences missing any CDRs and those from unhealthy human donors. The concept of diversity has been previously applied to other studies about the antibody repertoire.^20–22^

#### 2.1.2 Categorizing Sequences

The sequences were divided by chain and further into two subgroups: those produced by naïve B cells, and by memory B cells. Sequences of IgM antibodies were excluded from the memory group because this isotype is generally associated with low specificity and low affinity.^23^ Each of these groups was clustered at 95% identity using Linclust to eliminate redundancies, and ANARCI was used to number the sequences with the Martin scheme and determine their germline.^24–26^

#### 2.1.3 Determining the Gamma Diversity of CDRs

To quantify the comparative diversity of each CDR, we applied the concept of γ diversity, which measures the total diversity across an entire population.^20,21,27^ In this context, each CDR type was treated as a distinct population, defined by the complete set of its observed sequences. More details about this procedure can be found in the Supporting Information.

#### 2.1.4 Determining the Alpha Diversity of CDR Positions

To quantify the variability within individual positions of each CDR of memory antibodies, we applied the concept of α diversity, which measures the average diversity within individual subgroups of a population^20,21,27^ —in this case, specific sequence positions within CDRs of defined length and type. More details about this procedure can be found in the Supporting Information.

#### 2.1.5 Analyzing Framework Regions

Similarly to the CDRs, the α diversity of each framework position was calculated to identify the least conserved positions within the germline V region. The mutation frequencies of each of these positions in the memory antibodies were determined by aligning the sequences to their corresponding IMGT germlines using AbAlign.^28,29^ Analyses were repeated with the Murphy10 reduced amino acid alphabet to identify framework positions tolerant to class-changing mutations, such as SER to GLU.^30,31^ More details are available in the Supporting Information.

#### 2.1.6 Building Optimization Data Sets

After these analyses, we used the framework and CDR sequence data sets as the basis for the new data sets that Ab-SELDON uses in its different optimization steps. The first is a Naïve OAS CDR sequence data set (N-CDR), clustered at 40% identity to produce a much smaller but diversified pool of 1,129,369 sequences, enabling a broad paratope chemical space exploration with less epitope bias than memory antibody sequences.

The other data set is used by Ab-SELDON in the optimization of framework regions, which can significantly improve antigen binding.^32^ The Memory Framework data set (Me-FWK) comprises all 335,145 memory sequences from healthy donors. Its Mutated Framework subset (Mu-FWK) includes 231,932 sequences, all with at least four framework mutations compared to the IMGT germline, consistent with the minimum found in FDA-approved antibodies.^33^ In this subset, at least one mutation must be in a less conserved position impacting CDR conformation or the VH-VL angle, such as the Vernier regions or chain interface (Table S2).^32,34,35^

### 2.2 Optimization Pipeline Description

#### 2.2.1 Pipeline Overview

Starting from an input Ab-Ag complex and an antibody FASTA sequence, the Ab-SELDON pipeline optimizes the antibody through iterative modification cycles (Figure 1A). Each cycle involves probabilistically selecting a modification site, altering the antibody sequence, modeling the modified antibody, and scoring the modified Ab-Ag complex. Although Ab-SELDON requires a starting Ab-Ag complex structure, this input can originate from either experimental data (e.g., in affinity maturation scenarios) or computational methods such as docking or modeling, as is commonly done in de novo antibody design. The starting structure is not required to follow any particular antibody numbering scheme.

**Figure 1:**
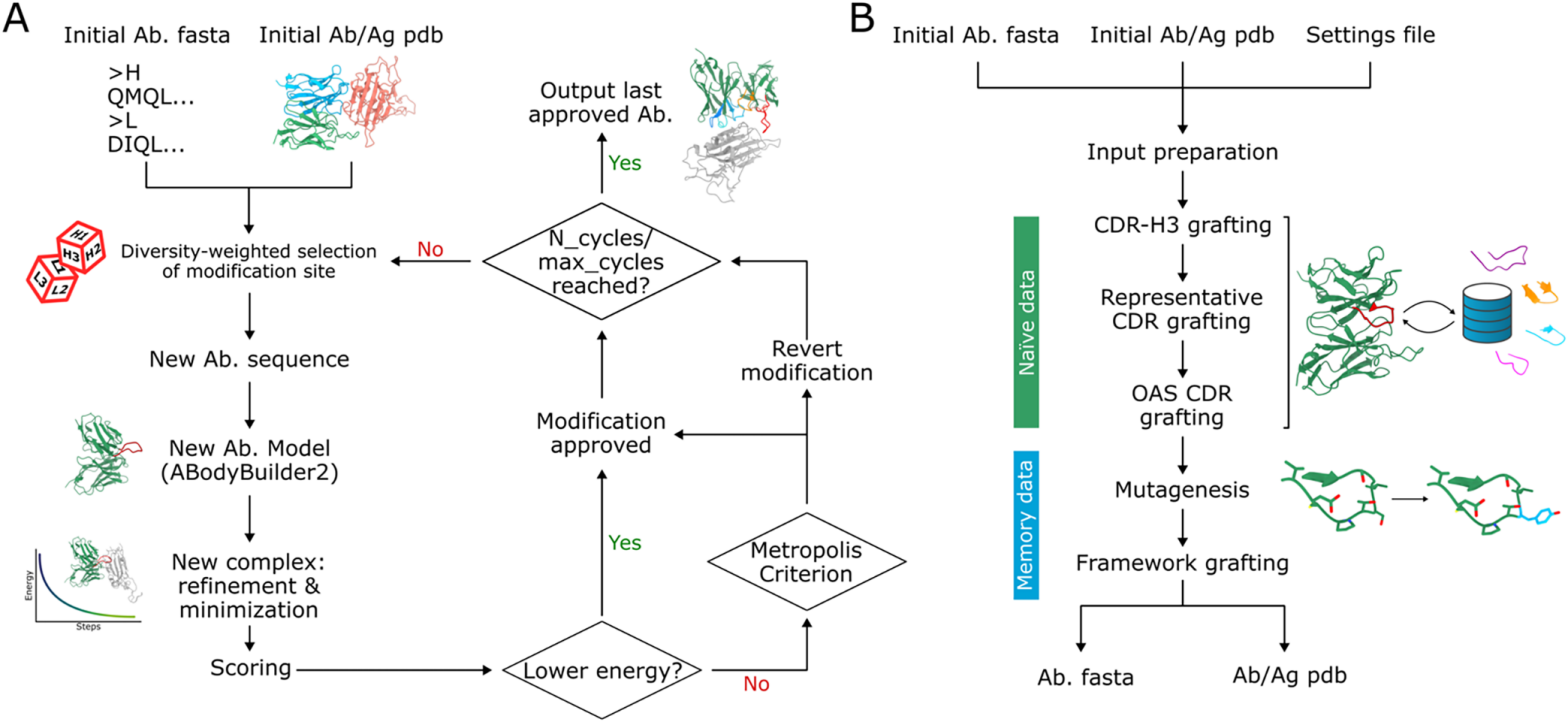
(A) Basic Ab-SELDON modification cycle performed in the CDR grafting and mutagenesis steps, assuming the use of the Metropolis Criterion for the approval or rejection of mutations. (B) Ab-SELDON’s default order of modification steps.

#### 2.2.2 Ab-SELDON Modification Cycle

By default, the program uses the diversity values of each CDR or position to determine the probability of a site being selected for modification, but users can set custom probabilities.

Then, a modification is chosen for that site using data sets of possible CDR sequences or mutations, with the specific data set depending on the current optimization step. The data sets used in the CDR grafting steps come from naïve antibodies, while the mutagenesis and framework optimization steps use data from memory antibodies, mimicking V­(D)­J recombination and somatic hypermutation, respectively.

The modified sequence is modeled using ImmuneBuilder’s ABodyBuilder2.^36^ The resulting model is then aligned to the complex using PyMOL, replacing the previous antibody.^37^ This is done to preserve the starting Ab-Ag interaction pose and reduce the computational cost of each cycle. The modified complex is subjected to an energy minimization using the Amber suite and scored using either CSM-AB or Rosetta’s REF15 scoring function, depending on the user’s choice.^38–42^

The well-established REF15 energy-based score is the default, because of its low computational cost and for having the highest accuracy for Ab-Ag evaluations among physics-based scoring functions, as shown in a previous benchmark.^41,42^ As an alternative, CSM-AB—a machine-learning-based method that builds upon earlier models such as mCSM-AB, mCSM-Ab2, and mmCSM-AB—offers higher accuracy than physics-based scorers but is slower, as it is accessed through a web server via an API.^40^ Further details of these modeling, minimization and scoring procedures can be found in the Supporting Information.

Based on the score of the new complex, the proposed modification is either accepted (with the modified antibody becoming the basis of the next cycles) or rejected using the Monte Carlo Metropolis criterion by default.^17^ However, two stricter criteria are implemented, approving only mutations that decrease the predicted interaction energy or those that reduce it by a user-customizable minimum threshold.

#### 2.2.3 Pipeline Optimization Steps

Users can allocate the modification cycles across five modular optimization steps, each targeting different aspects of the antibody optimization process. By default, the pipeline runs all five modules in a sequence that mirrors the biological progression from V­(D)­J recombination to somatic hypermutation in both CDRs and framework regions. However, users can run any number of modules in any order. This involves adjusting the pipeline’s configuration file, which also allows customization of numerous settings, most with predefined defaults (Figure 1B).

Before the start of the optimization process, a preparation step is run, which models the input antibody sequence and uses the resulting model to replace the antibody structure in the input Ab-Ag complex. This complex is used to calculate the score of the initial antibody. Additionally, this script also uses ANARCI to determine the V germline of the starting antibody and to number the sequence with the Martin scheme. The Martin scheme was chosen for its accuracy in tasks involving antibody structures, and was used as reference to define the CDR and DE loop regions.^34^

##### 2.2.3.1 CDR H3 Grafting

By default, the initial optimization step is CDR H3 grafting, as this CDR is typically the most variable and crucial for Ab-Ag interactions.^43,44^ In this module, naïve CDR H3 sequences from the previously described N-CDR data set are grafted onto the antibody. Based on the user-selected mode, it either tests H3 sequences of similar length to the starting antibody (default) or tests H3 sequences of any length present in the data set, either randomly or weighted by a distribution of CDR H3 lengths derived from the N-CDR data set.

##### 2.2.3.2 Grafting Representatives of Canonical Conformations of Non-H3 CDRs

Next comes the grafting of sequences representing the 53 canonical conformations of non-H3 CDRs from the PyIgClassify 2 database. The sequences were obtained from the median antibody structure of each canonical conformation, as specified in PyIgClassify2. When the median antibody was not human, the sequence was taken from the human antibody with the lowest dihedral distance from the median structure.^45^ As the PyIgClassify2 database uses the AHo numbering scheme to define the CDR regions, this step uses expanded CDR definitions that fit this database’s sequences for the grafting process (Table S3).

These 53 canonical representative sequences are iteratively grafted, modeled and scored. If a candidate conformation is accepted, it becomes the new reference, and the remaining alternatives are retested against this updated model. This cycle continues until none of the other 52 canonical structures yield further improvement. For CDRs H2 and L1, the DE loop is also cografted due to its impact on their conformation.^46,47^ This ensures that the influence of a CDR’s conformation on neighboring loops^48^ is taken into account and allows a comprehensive exploration of the CDRs’ conformational space within a limited number of cycles.

##### 2.2.3.3 Grafting of Non-H3 Naïve CDR Sequences

With the optimal paratope conformation chosen, the antibody undergoes a final grafting step where naïve sequences from the N-CDR data set are grafted onto non-H3 CDRs. By default, only CDR sequences that belong to the same V germline as the starting antibody and that, upon modeling, adopt the same conformation selected in the previous step will be tested. Whenever CDR H2 or L1 is chosen for grafting, the DE loop is also cografted. These constraints allow a greater exploration of the plausible chemical space for that same conformation. However, these germline and conformational requirements can be optionally disabled.

To simulate insertions and deletions, a customizable probability is used to determine whether the tested sequence will have either the same length, one more residue, or one less residue than the initial CDR, with default probabilities based on a previous study.^49^

##### 2.2.3.4 Mutagenesis

After all CDR grafting steps, by default, a mutagenesis step module introduces point mutations. The site of the mutation and the new residue are chosen based on diversity data from memory antibodies, although a random mutagenesis option exists. Mutations altering CDR conformation are avoided, unless the user permits them.

##### 2.2.3.5 Framework Grafting

In the final antibody modification step, the goal is to mature the non-CDR regions, since mature frameworks are associated with antibodies of higher affinity and specificity.^33,46,50^ The two chains of the antibody are aligned with the user-chosen set of memory framework sequences (Me-FWK data set or Mu-FWK subset) using BLAST to identify the closest matches.^51^ Hybrid antibodies are then generated, comprising one chain with a new mature framework and another with the original framework, while keeping the optimized CDRs. By default, only modifications preserving the paratope conformation are permitted.

The mature chains of these hybrids are combined to produce fully “mature” antibody variable regions, rechecking paratope conformations. Upon complex assembly, minimization, and scoring, the antibody with the lowest interaction energy is considered optimized and delivered as the final output if it improves upon the pre-framework graft antibody. Otherwise, the pre-framework graft antibody is delivered as the final output.

### 2.3 Pipeline Testing

#### 2.3.1 Evaluation of Scoring Protocol with SKEMPI

We evaluated the scoring protocol of the pipeline (comprising modeling, minimization, and scoring) using mutations documented in the SKEMPI 2.0 database.^52^ More information about the composition of this data set is available in the Supporting Information. We benchmarked the performance of Ab-SELDON’s default scoring function (REF15) with other state-of-the-art, deep-learning-based metrics. For this, in addition to REF15, a series of metrics calculated by AF3Score (pLDDT, pTM, iPTM, PAE and PAE Interaction)^53^ and ipSAE (ipSAE, pDockQ and pDockQ2)^54^ were used to evaluate mutated and wild-type structures produced by two approaches: the Ab-SELDON protocol, and a baseline protocol. AF3Score was used to apply AlphaFold 3 confidence metrics to these input structures without remodeling or altering their conformations. The REF15 predictions resulting from these two approaches were also compared using increasingly strict thresholds for approval of mutations.

In the Ab-SELDON approach, the mutations were applied to the antibody FASTA sequences, followed by structural modeling and alignment to the complex using the previously detailed protocol (Figure 1A). The wild-type antibodies were also subjected to the same protocol, allowing a better comparison of the changes predicted by the scoring functions.

For the baseline approach, we also performed an independent analysis using PyMOL’s mutagenesis wizard to introduce mutations directly onto the PDB-originated structures of the antibody–antigen complexes while preserving the original conformations and packing of the other residues. Both the wild-type and mutant structures were then evaluated by the scoring functions. This provided a baseline measurement of their predictive accuracy, isolated from perturbations introduced by modeling, alignment, and energy minimization.

Mutations were classified as correctly predicted if increased affinity in SKEMPI corresponded to a predicted improvement by the scoring function, like a decreased REF15 interaction energy or increased pLDDT, and vice versa. We also tested the performance of consensus metrics, where two scoring functions had to approve the mutation, leading to its rejection otherwise.

For the 40 mutations with available ΔΔ*H* data, we assessed the correlation between the experimental ΔΔ*H* and the change of the values predicted by REF15, AF3Score and ipSAE using Ab-SELDON’s protocol. All tests with this protocol were conducted in quadruplicate to account for variations introduced by the modeling and minimization processes.

#### 2.3.2 Comparison of Diversity-Guided and Random Optimization

We assessed whether using antibody diversity data could enhance the optimization process by comparing results from diversity-guided modifications to those from random modifications. Trastuzumab, a humanized antibody used for treating stomach and breast cancers,^55,56^ served as the modification starting point.

##### 2.3.2.1 Optimization Setup and Execution

The optimization runs were based on PDB structure 1N8Z.^57^ We conducted 16 optimization runs, equally divided between using and not using diversity data for modification selection, with all other parameters set to default and REF15 as the scoring function. More details of this procedure are available in the Supporting Information.

##### 2.3.2.2 Quantifying Conformational Diversity

Each output structure from the optimization runs was compared within its group (diversity with diversity, random with random) to evaluate the conformational variation achieved by each approach. To quantify the structural differences, we used FP-Zernike to calculate the Euclidean distance, where higher distances indicated greater structural divergence between each pair of antibody structures within each group.^58^ This comparison was done using the “protein mesh (PM)” representation option.

#### 2.3.3 Evaluation of Antibody–Antigen Interaction Stability before and after Optimization

We sought to evaluate Ab-SELDON’s ability to enhance interactions with a target epitope in a more challenging design task, where neither the antigen nor the starting antibody had a crystallized structure on the PDB. For this, we redesigned a pre-existing antibody to interact with cancer-related protein Galectin-3 binding protein (Gal-3BP) and utilized heated molecular dynamics (hMD) simulations to assess the stability of the Ab-Ag interaction pre- and post-optimization. This protocol has proven effective in differentiating between real and decoy Ab-Ag complexes.^59,60^ More information about how this method was applied in this work can be found in the Supporting Information.

The antibody and antigen models were docked using the blind docking server ClusPro with its specialized antibody mode activated, allowing the generation of a variety of plausible starting poses for optimization.^61–63^ Representative complexes of the highest-scoring clusters underwent hMD simulations. The pose whose interface RMSD stayed below the 5 Å threshold the longest was selected for optimization. This pose was also compared with structures produced by AlphaFold 3.^13^ After optimization with Ab-SELDON, the final Ab-Ag complex was assessed via hMD simulations, done with four replicates.

We also sought to compare Ab-SELDON’s performance with other, currently available pipelines. For this, the same complex submitted as input to Ab-SELDON was also submitted as input for Rosetta Antibody Design, and the diffusion-based RFantibody. The pipelines were compared in their ability to stabilize an Ab-Ag complex, and their computational performance. All three pipelines were run using their default settings. For the computational performance test, each step of Ab-SELDON was executed independently to determine its runtime. More details of these optimization runs are available in the Supporting Information.

## 3 Results and Discussion

### 3.1 CDR Gamma Diversity Analysis Reveals Enrichment of CDRs L1 and H2 upon Maturation

The OAS antibody sequence collection and filtering produced two heavy-chain data sets (naïve: 6,509,559 sequences; memory: 730,219 sequences) and two light-chain data sets (naïve: 4,994,546 sequences; memory: 332,331 sequences). From these data sets, we quantified and compared the γ diversities of all human antibody CDRs in different maturation states (Table S4). This analysis revealed that CDR3 exhibited the highest variability in both chains and maturation states, which was expected since it originates from multiple gene segments during V­(D)­J recombination (Figures S1C and S2). In agreement with Glanville et al., we found that CDRs H2 and L1 showed greater diversity than H1 and L2, respectively.^64^ This pattern was observed in both the naïve (Figure S1A,D) and memory antibody data sets (Figure S1B,E).

Interestingly, comparing the naïve and memory data sets, the diversities of CDRs H2 and L1 seemed enriched compared to the other CDRs from their respective chains after the maturation process. The ratio between the diversities (*D*_CDR1_:*D*_CDR2_:*D*_CDR3_) of the light chain CDRs was 25:1:675 in naïve antibodies, compared to 29:1:143 in memory antibodies, while in the heavy chain, the ratios were approximately 1:2:1,020,356 in naïve antibodies, and 1:6:377 in memory antibodies.

The higher diversity and further enrichment observed in CDRs H2 and L1 upon maturation suggest that these CDRs are particularly important for antigen binding and recognition. This aligns with a computational study analyzing antibody structures from the PDB, which concluded that CDRs H2 and L1 play a greater role in antigen recognition than CDRs H1 and L2.^65^

The enrichment of these CDRs post-maturation is likely due to the greater variety of lengths previously reported for these CDRs compared to the other (non-H3) CDRs in their respective chains, allowing a wider variety of SHM-induced mutations and indels.^45,66^ Indeed, a recent phage display study observed a 20-fold improvement in binding affinity after varying the lengths of CDRs H2 and L1.^67^

Therefore, these results suggest that CDRs H2 and L1 should receive more attention in computational antibody optimization processes, especially during optimization steps analogous to somatic hypermutation.

### 3.2 Antibody Optimization Tests

#### 3.2.1 REF15 is Competitive with AF3-Based Metrics and Superior for Multipoint Mutations

We benchmarked Ab-SELDON’s default scoring function (REF15) against state-of-the-art AlphaFold 3-based metrics, along with hybrid metrics based on the consensus of more than one scorer. This was done using 551 antibody–antigen mutations from the SKEMPI database. Across all mutations, REF15 had a lower F1-score (0.40), but achieved the highest accuracy for affinity-increasing mutations (61.6%) compared to ipSAE (60.6%) and pLDDT (60.2%) (( Table S5).

However, pLDDT performed better for the complete set of mutations and the subset of affinity-decreasing mutations (62.1% vs 53.8%), yielding the highest overall F1-score (0.43) (Table S5). A consensus between REF15 and pLDDT further improved rejection of nonbeneficial mutations (81.2% accuracy for affinity-decreasing cases), albeit at reduced sensitivity to beneficial ones (40.3%), achieving a slightly lower F1-score than pLDDT alone (0.41).

For the subset of 161 multipoint mutations, REF15 achieved the highest F1-score among all metrics (0.49 vs 0.47 for pLDDT), balancing improved accuracy for affinity-increasing mutations (70.7% vs 60.0% for ipSAE) with comparable accuracy for affinity-decreasing ones (Table S6). When applying a −2 REU threshold for approval on this subset, REF15′s F1-score increases further (0.50) (Table S7), with the affinity-increasing accuracy remaining equal to the best among AF3-based metrics (60% for both REF15 and ipSAE). Additionally, this threshold made the affinity-decreasing accuracy become better than all other metrics but pDockQ (77.9% vs 84.7%), which had the worst affinity-increasing accuracy (41.4%) (Table S6). As in the full data set, the REF15/pLDDT consensus showed the best rejection of nonbeneficial mutations (91.5% accuracy) but reduced detection of beneficial ones (45%) (Table S6).

For the 40 mutations with available ΔΔ*H* data, REF15′s predicted interaction energy changes showed a moderate correlation with experimental values (*r* = 0.57) (Figure S3A), slightly lower than those of pLDDT (*r* = −0.69) and ipSAE (*r* = −0.60) (Figure S3B,C). However, Ab-SELDON’s binary approval/rejection strategy should reduce this limitation, particularly for modifications that cause large structural changes, such as CDR grafting. Additionally, REF15 had the best correlation for affinity-increasing mutations and was second only to pLDDT for affinity-decreasing cases (Figures S4A and S5B).

Overall, when comparing the pipeline’s default scoring function (REF15) with AlphaFold 3-based metrics, we observed that while it had a somewhat inferior performance to pLDDT and ipSAE when evaluating all mutations in aggregate, REF15 was substantially superior for the subset of multipoint mutations. This is particularly important, considering Ab-SELDON’s emphasis on large-scale modifications on the antibody, like CDR and framework grafting.

Additionally, the calculation of REF15 has a much lower computational cost than the alternative metrics tested, requiring less than 2 s per structure on a CPU, compared to ∼46 s on an NVIDIA RTX 4090 GPU for AF3Score, which also depends on a >620 GB local AlphaFold database. Nevertheless, a future implementation of AF3Score’s pLDDT calculation on Ab-SELDON can become an advantageous alternative scoring method for the mutagenesis step.

#### 3.2.2 Ab-SELDON’s Scoring Protocol Improves Identification of Affinity-Increasing and Multipoint Mutations

We compared Ab-SELDON’s scoring protocol with a baseline approach using data from SKEMPI, assessing their ability to approve affinity-improving mutations and reject affinity-decreasing ones. Both used REF15 as the scoring function.

Across all 551 antibody–antigen mutations, Ab-SELDON’s protocol performed slightly worse overall than the baseline (56% vs 67%) (Tables S8 and S9). However, within the 132 affinity-increasing cases, Ab-SELDON substantially outperformed the baseline (62% vs 44%). Furthermore, Ab-SELDON was able to correctly classify more than 50% of both affinity-increasing and affinity-decreasing mutations, a balance that the baseline method failed to achieve under any threshold and which is reflected in its lower F1-scores. This difference became more pronounced with stricter approval thresholds.

For multipoint mutations, the baseline approved less than 30% of affinity-increasing mutations, while Ab-SELDON’s protocol not only increased its accuracy for these mutations to 71%, but also improved its identification of affinity-decreasing mutations to 67% (Tables S7 and S10). The baseline’s accuracy imbalance on the classification of the two types of mutations resulted in its substantially lower F1-scores and worsened sharply with the stricter approval thresholds. In contrast, Ab-SELDON’s accuracy and F1-score remained stable or improved slightly up to the −2 REU threshold (Table S7). Therefore, by tuning the approval threshold, users can emphasize detection of either affinity-increasing or affinity-decreasing modifications while preserving overall classification balance and performance.

Taken together, this suggests that Ab-SELDON’s modeling and minimization protocol better captures post-modification conformational changes that enable improved Ab-Ag interactions, though it has difficulty identifying detrimental mutations that cause steric clashes. An improved performance for both types of mutations was observed when evaluating multipoint mutations, showing the protocol’s ability to cope with larger-scale changes to the antibody.

While these results suggest that Ab-SELDON’s protocol for modeling and scoring mutations has a similar or superior performance when compared to a baseline approach, its performance remains constrained by the accuracy of existing scoring functions, including REF15 and even more modern deep-learning-based metrics. This limited success may stem from the higher flexibility inherent to Ab-Ag interactions, which can be difficult to account for when scoring static structures.^42,68^ This could lead to beneficial mutations getting rejected and detrimental ones getting approved during optimization.

However, the scoring protocol was able to accurately evaluate the effectiveness of a majority of proposed mutations, especially multipoint and those that improve binding affinity, with this effect being more pronounced when using a stricter threshold for approval. Therefore, it is expected that, after a sufficient number of modification cycles, Ab-SELDON’s optimization process should lead to a net improvement of the Ab-Ag interaction.

#### 3.2.3 Diversity-Guided Optimization Leads to More Conformationally Diverse Antibodies

To assess the performance of the pipeline in antibody optimization tasks, we applied Ab-SELDON to further optimize trastuzumab, a humanized monoclonal antibody that targets the Human Epidermal Growth Factor Receptor 2 (HER2). Overexpression of HER2, involved in signaling pathways that drive cell proliferation, has been reported in several malignancies, including breast, lung, and gastric cancers.^69^ Anti-HER2 antibodies such as trastuzumab have been successfully used to treat HER2-positive tumors by promoting receptor internalization and degradation.^70^ Owing to its clinical relevance and extensive structural characterization, trastuzumab has also served as a test case in multiple antibody design studies.^71–75^

In the optimization trials performed in this study, between 525 and 608 cycles were executed per run, due to the variable number of cycles in the representative CDR grafting step. These runs took between seven and 12 hours to complete.

We plotted the Ab-Ag interaction energy from trastuzumab modifications approved throughout all runs. In the modification steps where the diversity data could influence the modification selection process (representative and OAS CDR grafting, and mutagenesis), the diversity-guided runs saw, on average, slightly lower final interaction energy values and a slightly higher total number of modifications approved than randomized ones (146 for diversity versus 118 for random) (Figures S6 and S7). In this example, the framework grafting step resulted in no approved modifications, likely because trastuzumab’s framework regions, with 27 pre-existing mutations, were already near-optimal.^33^

To assess whether a diversity-guided approach enhances the conformational exploration of the antibody, each output structure from optimization runs was compared within its group (diversity with diversity, random with random). Results indicated that structures from diversity-guided modifications had significantly higher Euclidean distances, compared to randomized modifications (average values of 1.7 and 1.3 for diversity and random runs, respectively, *p* = 0.004). This suggests that a broader exploration of the antibody conformational space was achieved by the diversity-guided approach (Figure S8).

### 3.3 Ab-SELDON Improves Antibody–Antigen Interaction Stability at Lower Computational Cost

To evaluate Ab-SELDON, RAbD, and RFantibody in cases with limited structural information, the pipelines were used to optimize an anti-Gal-3BP antibody, starting from modeled antibody and antigen structures docked together. Gal-3BP is a soluble protein with a regulatory role in the immune system.^76^ Its overexpression is associated with a poor prognosis for many types of malignancies, including breast and lung cancers, where it is involved in numerous pro-tumoral mechanisms, like angiogenesis, migration, adhesion, motility, and immune response. This has led to the identification of Gal-3BP as a promising target for inhibition by immunotherapeutics.^77^

Because predicting antibody–antigen affinity from static structures remains challenging,^42^ we assessed pre- and post-optimization Ab-Ag interaction stability using heated molecular dynamics (hMD) simulations (details in Supporting Methods). In all simulations of the initial antibody-Gal-3BP complexes produced by ClusPro, the iRMSD exceeded 5 Å, particularly at higher temperatures. However, one complex, Clus2, maintained iRMSD below 5 Å for longer and at higher temperatures than the alternatives and was considered the most promising for optimization (Figure S9).

To further assess this starting structure, we compared it against models generated by AlphaFold 3 (AF3). Notably, AF3 predictions broadly agreed with the Clus2 binding region, with four out of five models targeting the same functional domain (Gal-3BP BTB/POZ domain, which mediates oligomerization, among other functions^78^) as ClusPro (Figure 2A,B). While this domain-level agreement reinforced the biological plausibility of the Clus2 pose as a robust starting point for refinement, the specific epitopes differed and the AF3 models themselves were assigned low confidence scores (best model: ipTM = 0.26; pTM = 0.51; ipSAE = 0.08). Therefore, the Clus2 complex was selected as the starting point for the optimization process, which was done using the default settings for all pipelines, including diversity-guided modifications for Ab-SELDON. The optimized complexes were then compared using new hMD simulations.

**Figure 2:**
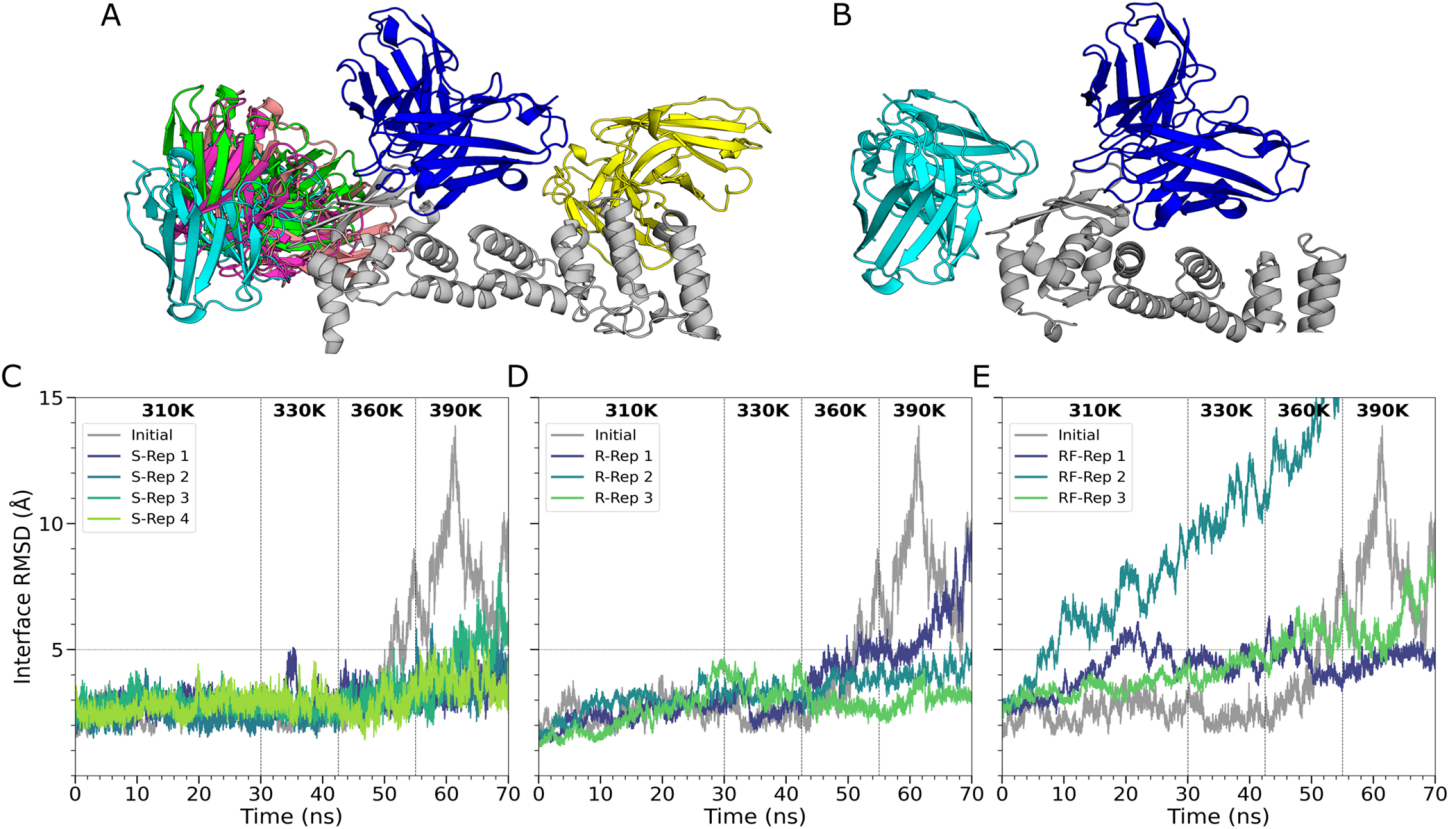
Comparison of Clus2 complex with (A) all AF3 complexes, and with (B) only the best AF3 complex. Antigen (gray), AF3 poses (cyan, green, salmon, magenta and yellow), and ClusPro pose (dark blue); iRMSD of the optimized antibody-Gal-3BP complexes during hMD replicates produced by (C) Ab-SELDON, (D) Rosetta Antibody Design, and (E) RFantibody.

In the case of Ab-SELDON, the simulations revealed that the optimized antibody exhibited more stable interactions with the antigen. Notably, except for a brief spike at 330 K, the iRMSD of replicate 1 (S-Rep1) stayed below 5 Å, even at the highest simulation temperatures (Figure 2C). Considering that Ab-Ag interface stability has been previously correlated with higher affinity,^79^ these results indicate that Ab-SELDON was able to effectively optimize the antibody paratope to stabilize the Ab-Ag interface, even after starting from modeled antibody and antigen structures.

The antibody optimized by RAbD also exhibited improved stability, with R-Rep3 remaining below 5 Å throughout the simulation, though R-Rep1 destabilized at high temperature, reaching 9.67 Å (Figure 2D). In comparison, Ab-SELDON’s least stable replicate (S-Rep3) peaked at 8.46 Å but partially recovered by the end.

None of the antibodies generated by RFantibody remained stable over the course of the simulations (Figure 2E), with replicate 2′s (RF-Rep 2) iRMSD crossing the 5 Å threshold within the first 10 ns and rapidly increasing throughout the simulation, suggesting a destabilization of the complex. These observations suggest that optimization by RFantibody did not substantially improve antibody–antigen interaction stability. We also compared the computational performance of the different pipelines (Table S11). Normalized per modification cycle (or structure for RFantibody), Ab-SELDON completed one cycle in 62.2 s, compared to 100.7 s for RAbD and 400 s for RFantibody, representing 38.2% and 84.4% shorter runtimes, respectively. Within Ab-SELDON, the CDR grafting and mutagenesis steps took similar times per cycle, while the framework step was slower (101.1 s/cycle), due to additional modeling events.

Together, these results demonstrate that Ab-SELDON achieves comparable or superior stabilization of antibody–antigen complexes while operating with lower computational cost than existing antibody design pipelines. Although activating docking in RAbD or increasing structure generation in RFantibody could improve their performance, such changes would substantially increase runtime.

## 4 Conclusion

The increasing significance of monoclonal antibodies in treating cancers, infectious diseases, and autoimmune disorders has driven the demand for user-friendly and computationally efficient in silico antibody design tools. In this context, our proposed Ab-SELDON pipeline, designed for modularity and ease of use, employs a diversity-driven approach to optimize antibody structures, leveraging human antibody sequence and conformational variability data to enhance Ab-Ag interactions. By integrating ABodyBuilder2, a modern deep-learning-based modeling tool, with Amber’s GPU-based energy minimization feature, Ab-SELDON is able to accurately generate and evaluate modified antibody structures. For a small antigen, each modification cycle takes under 1 min on a laptop GPU (NVIDIA RTX 3060 Mobile) and approximately 30–40 s on a workstation GPU (NVIDIA RTX A6000).

During trastuzumab optimization testing, Ab-SELDON’s diversity-guided modifications achieved slightly lower Ab-Ag interaction energies and produced more conformationally diverse antibodies compared to randomized modifications.

Additionally, tests using SKEMPI’s experimentally measured Ab-Ag mutations indicated that Ab-SELDON’s modeling and minimization protocol accurately predicted the effects of the majority of mutations, especially multipoint and those that increased affinity. The pipeline’s default scoring function (REF15) was also shown to have a competitive performance when compared to state-of-the-art metrics based on deep learning, and at a far lower computational cost.

The performance of the pipeline was also compared with currently available alternatives, both classic (Rosetta Antibody Design) and diffusion-based (RFantibody), by comparing their ability to improve an initially unstable complex produced through modeling and docking of the Ab and Ag structures. Heated molecular dynamics of the optimized complexes showed that Ab-SELDON was able to produce similar or better results at a lower computational cost than the alternatives. These results demonstrate Ab-SELDON’s ability to efficiently design antibodies with improved predicted interaction energy and stability against target antigens, proving its utility in antibody engineering.

The pipeline’s performance and accuracy are limited by the currently available scoring functions and by the low availability of data on human memory antibodies (relative to naïve antibodies) on OAS. However, the modular nature of the pipeline’s algorithm simplifies the integration of future tools that can improve it in these areas. Planned upgrades include the integration of DL-based scoring functions, such as AF3Score’s pLDDT and ipSAE, that can more accurately evaluate antibody mutations, albeit at an increased computational cost; and of an antibody-specific Language Model with low germline bias that can favor mutations that lead to higher affinity and specificity, such as AbLang-2.^19^

## Supplementary Material



## Data Availability

The source code, optimization data sets and user instructions are freely available at https://github.com/SFBBGroup/Ab-SELDON. The testing data is available at 10.5281/zenodo.15066729.

## Notes

Abseldon information contains additional information about the methods used in the article, as well as additional figures and tables (PDF)

## Abbreviations

Ab: antibody
Ag: antigen
Ab-SELDON: Antibody Structural Enhancement Leveraging Diversity for Optimization of iNteractions
