# Supplementary material for: Ab-SELDON: Leveraging Diversity Data for an Efficient Automated Computational Pipeline for Antibody Design
Source: J Chem Inf Model. 2026 Jan 20;66(3):1895–905. doi: 10.1021/acs.jcim.5c01924 (PMC12892330; doi:10.1021/acs.jcim.5c01924)
Supplement: Supplementary file 1 [file ci5c01924_si_001.pdf]

Ab-SELDON: Leveraging diversity data for an efficient automated computational pipeline for antibody design

Jean V. Sampaio[1,2], Andrielly H. S. Costa[1,2], Aline O. Albuquerque[1], Júlia S. Souza[1,3], Diego S. Almeida[1,2], Eduardo M. Gaieta[1,2], Matheus V. Almeida[1,3], Geraldo R. Sartori[4,], João H. M. Silva[1,2,5,*]*

[1]Laboratory of Structural and Functional Biology Applied to Biopharmaceuticals, Fundação Oswaldo Cruz, Fiocruz Ceará, Eusébio, 61773-270, Brazil.

[2]Instituto Oswaldo Cruz, Fiocruz, Rio de Janeiro, Rio de Janeiro, 21040-900, Brazil.

[3]Universidade Federal do Ceará, Fortaleza, Ceará, 60020-181, Brazil

[4]São Carlos Institute of Physics, University of São Paulo, São Carlos, São Paulo, 13566-590, Brazil.

[5]Pasteur-Fiocruz Center on Immunology and Immunotherapy, Fundação Oswaldo Cruz, Fiocruz Ceará, Eusébio, 61773-270, Brazil.

*Corresponding authors: joao.martins@fiocruz.br (J.H.M.S.), geraldo.sartori@ifsc.usp.br (G.R.S.)

Supplementary methods

Calculation of CDR gamma diversity

To determine the degree of variability in individual human CDRs, the CDR sequences from OAS antibodies were initially categorized by type (H1, H2, H3, L1, L2, and L3) and by maturation state (naive or memory). From the set of numbered complete sequences, only the residues corresponding to the CDRs, based on Martin numbering, were extracted in fasta format. The CDR definitions used here can be found at the S2 Table.

The sequences of each CDR type were then clustered at 100% identity using the Linclust software to group identical CDR sequences, including repeated CDRs even in non-redundant antibodies.

The number and size of the clusters generated for each CDR type were analyzed. For each CDR, the number of clusters produced corresponded to the number of unique CDR sequences found in non-redundant antibodies. The size of these clusters, in turn, reflected the number of times a particular CDR sequence was found in the non-redundant antibodies.

These cluster characteristics were used to calculate the gamma diversity of each naive and memory CDR type, based on Shannon's entropy (*H*). Using equation 1, for each CDR type, Shannon's entropy (H) was derived from the negative sum of the frequencies of each unique CDR sequence (p_i) within the set of non-redundant sequences of that CDR type, multiplied by the logarithm of those frequencies.

$$H = -\sum_{i}^{k} p_i \log p_i$$

Equation 1. Equation for the calculation of Shannon's entropy. *H* = Shannon's entropy; k = number of distinct outcomes; p_i = fraction of population composed of a single species i.

Thus, a CDR with many small clusters—i.e., with several sequences that are repeated only a few times—would have a high sequence variety and, therefore, high entropy, and vice versa. Based on Shannon's entropy, the true diversity of each CDR was calculated, allowing quantitative comparisons between the obtained values [1]. This was done using equation 2, where D represents the true diversity value.

$$\mathrm{D} = \exp(H)$$

Equation 2. Equation for the calculation of true diversity (based on Shannon's entropy). The true diversity (D) is the exponential of Shannon's entropy (*H*).

Calculation of CDR position alpha diversity

Starting from the filtered datasets of memory antibody CDR sequences, subsets were created by grouping the CDRs from these sequences that had the same length. For each of these subsets, the number of different residues and the frequency of each amino acid residue at each position of the CDR were used to calculate the alpha diversity of that position, using, again, equations 1 and 2, similarly to the calculation of CDR gamma diversity. This analysis allows the identification of the sites within each CDR type and length that are most prone to mutations during somatic hypermutation. For this calculation, only CDR type/length combinations with at least 100 sequences were considered. Additionally, the amino acid residues and their respective frequencies at each of these positions were identified.

The data was stored in CSV files, with columns containing, in this order, the position number, its alpha diversity, and the frequencies of each residue observed at that position in Python dictionary format. These CSV files are used in the mutagenesis step of the antibody modification program, where mutations are introduced more frequently at less conserved positions, and the most likely residues for each chosen position are tested.

Framework analysis

In order to identify the main mutations and maturation mutation sites in framework regions, the same set of filtered and numbered memory antibody sequences was used to identify these regions and their respective germline V gene segments. Thus, all OAS memory antibody sequences from healthy human donors analyzed were categorized according to their germline. This analysis was limited to the V segment.

Next, these sequences were aligned to their respective germline gene segments, extracted from the IMGT database [2]. This was done using the AbAlign program [3]. The program's default parameters were used for this alignment, specifying only the use of Martin's numbering. This alignment allowed a position-by-position comparison of the framework of matured sequences with their pre-maturation analogs.

After the analyses with the original sequences were completed, the analyses were repeated after converting these sequences to a reduced amino acid alphabet, in which residues with similar chemical characteristics are considered as one. The Murphy10 alphabet was chosen, which contains 10 amino acids instead of 20, because it is considered the smallest reduced alphabet with acceptable sensitivity in studies of homology, function prediction, and protein alignment [4–7]. This was done to better highlight framework positions capable of accepting a wide variety of residues, including those belonging to a different class than that of the original germline.

For each germline segment, the total number of differences found in its set of sequences at each position (compared to the original IMGT germline sequence) was divided by the total number of sequences to calculate the mutation rate at each position. The Shannon entropy (H) of the set of residues appearing at each position was also calculated. These entropies were then converted into true diversity.

The top 10% or top 20% of positions for each germline segment or germline family with the highest mutation rates and/or diversities were identified. Those whose mutation rates stood out in at least half of the germlines or germline families analyzed (for example, IGHV1, IGHV2, etc.) were selected. Separately, positions with high mutation rates and/or diversities in all or in all but one of the germlines were also identified.

Based on the obtained data (Supplementary Table 3), positions of interest for the framework grafting step of the modification routine were identified. With the exception of heavy chain residue 73—which is part of the DE loop co-transplant during the CDR grafting process—and residues at the interface between the variable and constant domains (Fv/c), all highlighted positions whose role in the antibody structure was corroborated by literature were selected as reference for the assembly of the framework sequence dataset. In the heavy chain, these residues were 35, 50, 57, 58, and 94. In the light chain, these residues were 2, 4, 43, 46, and 49 [8–14].

Framework sequence dataset

Based on these analyses, a database of memory antibody sequences was assembled to be used in the framework grafting step of Ab-SELDON. Based on previous studies showing that antibodies approved for therapeutic use have a minimum number of mutations in the framework [15] and demonstrating that highly affinity-matured antibodies exhibit greater structural rigidity [14,16,17], sequences were selected using the following criteria:

- At least 4 mutations in the framework relative to the original germline [15];
- At least one mutation in any of the framework positions with high variability that influences the conformation of CDRs, the angle between the light and heavy chains, the overall rigidity of the antibody, or the antibody/antigen affinity [11–13].

Another dataset, which includes all memory framework sequences (whether or not they obey these criteria) is also available to users. Due to the high inherent conservation of the framework regions, the framework sequences of both datasets were once again filtered using Linclust with a 100% identity filter.

Ab-SELDON’s scoring protocol

Modelling of the antibody structure within the pipeline is done using AbodyBuilder2, changing the numbering scheme to Martin and disabling the refinement of side chains [18].

```
predictor = ABodyBuilder2(numbering_scheme="martin")
```

check_for_strained_bonds=False

After aligning the new model with the original Ab-Ag complex using PyMOL, the previous antibody structure in the complex is removed, leaving only the antigen and new antibody structure in the pdb file [19]. This alignment procedure was chosen instead of docking or Ab-Ag complex modelling to both guarantee the preservation of the original orientation of the newly modified antibody in relation to the antigen, but also to avoid the higher computational cost associated with these methods.

This complex is submitted to AbodyBuilder2's side chain refinement function, correcting the packing of both antibody and antigen side chains, partially reducing the clashes generated by the structural alignment process.

from ImmuneBuilder.refine import refine

refine(new_complex.pdb)

This refined structure is then minimized using AmberMD, to further reduce clashes. For this, the complex is protonated with PDB2PQR's Propka algorithm using a user-specified pH value (default = 7) [20,21]. This protonated complex is parametrized in AmberTools with tleap, using the ff14SBonlysc force field [22]. Hydrogen mass repartition (HMR) is also performed using parmed [23]. Finally, the complex is submitted for energy minimization in pmemd using an implicit solvent (igb = 8). The minimization process is run until the system reaches convergence (maxcyc = 99999). The use of structural alignment, HMR and the implicit solvent reduces the compute time required for minimization to less than 30 seconds, depending on the GPU model used and the number of residues of the targeted antigen [24,25].

The minimized complex structure is submitted to CSM-AB for scoring, when this scoring function is chosen [26]. When using REF15, the energy of the whole complex and the antibody and antigen in isolation are calculated. Then, the final REF15 score is calculated by subtracting the energies of the isolated molecules from the energy of the whole complex, resulting in the antibody-antigen interaction energy, as detailed by Guest et al. [27,28]. For the application of the Monte Carlo Metropolis criterion, the kT constant is defined as 0.5 for CSM-AB (as it estimates the interaction energy in kcal/mol), and 1.0 for REF-15 (as this is the default value used by Rosetta Antibody Design in this step) [26,29]. Alternatively, the user may choose two other modification approval modes: "normal", where any increase in REF15/CSM-AB energy leads to the rejection of the modification; and "strict", where a minimum user-specified energy decrease is needed to approve the modification.

Evaluation of scoring protocol with SKEMPI

We evaluated the scoring protocol of the pipeline—comprising modelling, minimization, and scoring—using mutations documented in the SKEMPI 2.0 database [30]. A baseline, PyMOL-based protocol was also evaluated. We also benchmarked the pipeline's default scoring function (REF15) against a series of metrics calculated by the AF3Score (pLDDT, pTM, iPTM, PAE and PAE Interaction)[31] and ipSAE (ipSAE, pDockQ and pDockQ2) [32] softwares.

After the structures produced by both protocols were evaluated by AF3Score, the resultant CIF structures and the full JSON files produced by AF3Score were used as inputs for ipSAE. Because the ipSAE, pDockQ, and pDockQ2 metrics (as calculated by the ipSAE script) are each defined for a pair of interacting chains, we computed these metrics for the antigen interacting with the heavy and light antibody chains separately and combined them into a single antibody–antigen score using Equation 3.

$$M = \frac{\sum_{i \in \{\text{H-Ag, L-Ag}\}} M_i \times n_{0res,i}}{\sum_{i \in \{\text{H-Ag, L-Ag}\}} n_{0res,i}}$$

Equation 3. Aggregate interaction metric M for the antibody–antigen complex (where M is ipSAE, pDockQ, or pDockQ2), computed as the weighted average of pairwise values M_i; here M_i is the metric for antigen–chain pair i (H–Ag or L–Ag) and $n_{0\text{res},i}$ is the number of inter-chain residue pairs that passed the PAE and distance cut-offs.

Each pairwise metric M_i was taken from the "max" result reported by the ipSAE program for that antigen–chain pair (i.e., the larger of the two asymmetric direction scores reported for the pair). We then combined the antigen–heavy chain (H–Ag) and antigen–light chain (L–Ag) contributions using equation 3, weighting each pairwise metric by the number of inter-chain residue pairs $n_{0\text{res},i}$ that satisfied the PAE and distance thresholds. This yields a single representative score M for the full antibody–antigen interaction while accounting for the relative interface sizes reported by the program.

Because of the generally lower confidence of Ab-Ag predictions by AlphaFold-based metrics (when compared to other PPIs), ipSAE was run with a PAE cutoff of 32 and a distance cutoff of 15. PAE cutoffs of 10-15, recommended by the original ipSAE study[32], generally resulted in ipSAE values equal to zero between the antibody and antigen chains, even for unaltered Ab-Ag complex structures taken from the PDB. Therefore, all ipSAE calculations in this work were performed with the described protocol and parameters.

Of the 563 available single and multipoint mutations introduced into the antibody chains of Ab-Ag complexes across 30 distinct PDB structures, we analyzed the 551 that had

either increased or decreased the antibody's affinity. Of these, 132 increased, and 419 decreased binding affinity. Of the 161 multipoint mutations, 35 increased, and 126 decreased binding affinity.

The Ab-SELDON protocol tests were done in quadruplicate, resulting in n=2204 for all mutations (4*551), n=644 for multipoint mutations (4*161), and n=160 for the 40 mutations with ΔΔH information. Because of the low variation observed in the PyMOL-based threshold tests and the high computational cost of AF3Score (~46s per structure evaluated, using a NVIDIA RTX 4090 GPU), only one replicate (n=551) was used for the scoring function benchmark done with the baseline protocol.

Anti-HER2 antibody optimization setup and execution

The optimization runs were based on the PDB structure 1N8Z, which consists of trastuzumab in complex with HER2 [33]. The waters, ions and other heteroatoms were removed, along with the constant regions of the antibody (i.e. only the variable portion of the antibody was used). However, this structure has a missing loop in the HER2 structure, corresponding to residues 580-591, which are part of trastuzumab's epitope.

To address this gap, the PDB structure 8FFJ [34], which includes HER2 bound to a different antibody and contains the missing loop, was aligned with 1N8Z using PyMOL's align function. This alignment put the extremities of 1N8Z's gap near the extremities of the aligned loop. Subsequently, all residues from 8FFJ were excluded except for those of the missing loop. Two covalent bonds were then added to fuse the loop with the HER2 backbone in 1N8Z using PyMOL's fuse function:

1) A bond between the amide nitrogen atom of residue 593 in 1N8Z and the carbonyl carbon atom of residue 614 in the loop;

2) A bond between the carbonyl carbon atom of residue 578 in 1N8Z and the amide nitrogen atom of residue 601 in the loop.

Finally, in the resulting structure, all HER2 residues outside the epitope region (residues 486-607) were removed, as they do not interact with trastuzumab. Ab-SELDON received this final structure as input and was executed with default settings.

Anti-Gal-3BP antibody modelling, docking and optimization

The model of a pre-existing antibody called SP-2[35] were created with AlphaFold 2 and ABodyBuilder 2, as well as the IgFold and DeepAB[18,36–38]. The model of Gal-3BP (comprising residues 20-405 of Uniprot[39] entry Q08380) was created with AlphaFold 2. All models were generated using the default parameters suggested by the developers. The quality of the antibody models was evaluated using their pLDDT values and the VoroMQA program[40].

Based on the comparative modeling values of the SP-2 antibody, the structure generated by AlphaFold 2 was selected for the binding mode prediction with Gal-3BP. For this purpose, the ClusPro blind docking server was used with the antibody mode activated. ClusPro was chosen for being a well-established method with a good performance on Ab-Ag interactions among blind docking programs, and with a specialized antibody docking mode [41–43]. This allowed the generation of varied, but plausible, candidate poses for optimization. The AlphaFold 3 Server[44] was also tested for this purpose, but the generated complexes were of low confidence (best model: ipTM = 0.26; pTM = 0.51; ipSAE = 0.08) and, therefore, not selected for further testing or optimization.

The most stable pose (assessed with Heated Molecular dynamics; details on the next section) was selected for optimization. Optimization tests were done for Ab-SELDON using default settings.

As this main optimization run with Ab-SELDON took a total of 567 modification cycles, this was also the number of outer cycles specified for the optimization run with Rosetta Antibody Design, and the number of designs specified for RFantibody. Both programs were run with default settings (in the case of RAbD, this meant that no dockings were performed during optimization. RAbD was executed with MPI enabled). To ensure the results from this pipeline could be properly compared to the alternatives tested, RFantibody was provided with the model of the SP-2 antibody as the starting framework, through the option *antibody.framework_pdb*.

In the case of RFantibody, the complex with the lowest PAE among the generated structures was chosen for evaluation with hMDs. Due to missing peptide bonds, Modeller's[45] AutoModel function was used to refine this complex, producing 1000 structures. The structure with the lowest molpdf value was used as input for RFantibody's hMD simulations.

Separately (and only for the runtime comparison), Ab-SELDON's modules were executed individually using the same settings and hardware as the other optimizations, to determine the runtime of each individual step. Because of the variability in the number of cycles executed by the Representative CDR Grafting step, the total number of modification cycles executed in this test was 669. The runtime comparisons were therefore done using a time per cycle/generated structure metric. In the case of RFantibody, the time per generated structure includes the RFDiffusion, ProteinMPNN and RF2 steps.

Anti-Gal-3BP antibody molecular dynamics

To evaluate the complexes generated by the docking program and the complexes outputted by Ab-SELDON, RFantibody and Rosetta Antibody Design, they were subjected to Heated Molecular Dynamics (hMD)[46,47] using the Amber20 software package [21], employing the FF19SB protein force field and OPC water model [48,49]. These simulations, as described by Chaves et al., are performed in four parts. The system starts

at a temperature of 310 K in the first step, increasing to 330 K, 360 K, and finally 390 K in the three following parts of the simulation, with each four-step simulation spanning a total of 70 ns. The Ab-SELDON hMDs were performed in quadruplicate, while those for the other pipelines were performed in triplicate.

To calculate the protonation state of the residues, PDB2PQR was used at a pH of 6.8 [20]. The complexes were centered in an octahedral box with a 10 Å buffer from the edge, and the system was neutralized with Na^+ or Cl^- ions. To prepare the topology and coordinate files for the production dynamics, the system was subjected to a relaxation process divided into 10 steps [50,51]. Initially, the water was minimized for 1000 steps using the steepest descent method, followed by 9000 steps using the conjugate gradient method, with weak restraints of 1 kcal/(mol·Å^2) applied.

Next, the system was heated to 310 K under constant volume (NVT) over 0.5 ns, applying positional restraints of 100 kcal/(mol·Å^2) to all atoms except hydrogens and water molecules. The equilibration of the water density was carried out with restraints of 100 kcal/(mol·Å^2) over 1 ns under a constant pressure of 1 bar. Subsequently, the restraints were reduced to 10 kcal/(mol·Å^2) and the system was equilibrated for another 1 ns. Then, a second minimization was performed for 10,000 steps using the conjugate gradient method, with restraints applied only to the main chain atoms using a force constant of 10 kcal/(mol·Å^2).

The system was then gradually released in three successive stages, each lasting 1 ns, under constant pressure and temperature conditions (NPT). Positional restraints on the backbone atoms were gradually reduced from 10 kcal/(mol·Å^2) to 1 kcal/(mol·Å^2) and finally to 0.1 kcal/(mol·Å^2). The equilibration protocol was completed with 1 ns of unrestrained molecular dynamics (MD), also under constant NPT conditions. The cutoff radii for Coulomb interactions, Lennard-Jones interactions, and the cutoff distance for the short-range neighbor list were standardized to 8 Å.

The production dynamics were carried out with the CUDA version of Partial Mesh Ewald Molecular Dynamics (PMEMD) [52] and with the application of Hydrogen Mass Repartitioning (HMR), which allowed the simulation to be performed with a timestep of 4 fs [23]. Temperature and pressure were controlled using the Langevin thermostat and the Monte Carlo barostat. As defined for the heated simulation [46], the temperature was maintained at 310 K for the first 30 ns; then, for the following 12.5 ns, it was raised to 330 K, followed by another increase to 360 K; the last 15 ns of the simulation were conducted at 390 K. CPPTRAJ, implemented in the Ambertools 22 package [53], was used for analyses of interface RMSD (iRMSD), contact evaluation, hydrogen bonds, among other assessments.

System details

The Ab-SELDON pipeline requires the following software:

Python (tested versions: 3.11.6; 3.12.3)

AmberMD and AmberTools (tested versions: Amber 22; Amber 24. Note: CUDA and PMEMD must be enabled)

ImmuneBuilder (tested versions: 1.0.1; 1.1.1)

PyRosetta (tested versions: pyrosetta-2023.36; pyrosetta-2024.19)

PDB2PQR (tested version: 3.6.1)

ANARCI

BLAST (tested versions: 2.12.0; 2.15.0)

pdb-tools (tested version: 2.5.0)

PyMOL (tested versions: 2.6.0a0; 3.0)

Optimization runs in this article used NVIDIA RTX A6000 GPUs (48 GB VRAM), but the pipeline has previously been run successfully using an NVIDIA RTX 3060 Mobile GPU (6 GB VRAM). Scoring tests with AF3Score were done using an NVIDIA RTX 4090 GPU (24 GB VRAM).

Supplementary figures and tables

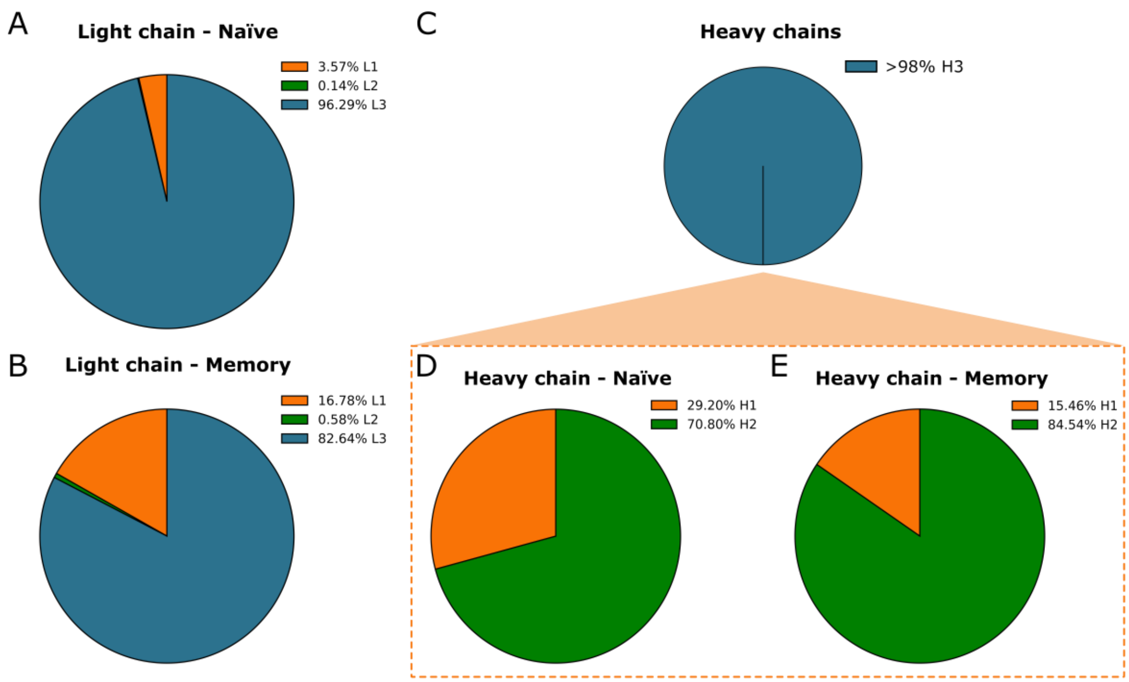

Supplementary Figure 1. Proportions between the gamma diversities of the different CDRs in the light chains of (A) naïve and (B) memory antibodies; and of (C) H3 and non-H3 CDRs in the heavy chains of (D) naïve and (E) memory antibodies.

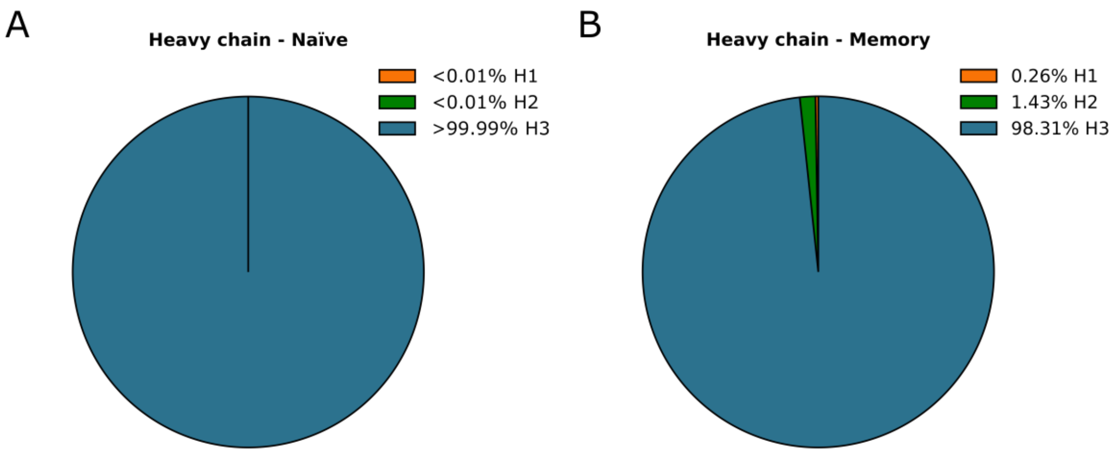

Supplementary Figure 2. Proportions between the gamma diversities of the different CDRs (including CDR H3) in the heavy chains of (A) naïve and (B) memory antibodies. For both chains, CDRs 1, 2 and 3 are in orange, green, and blue, respectively.

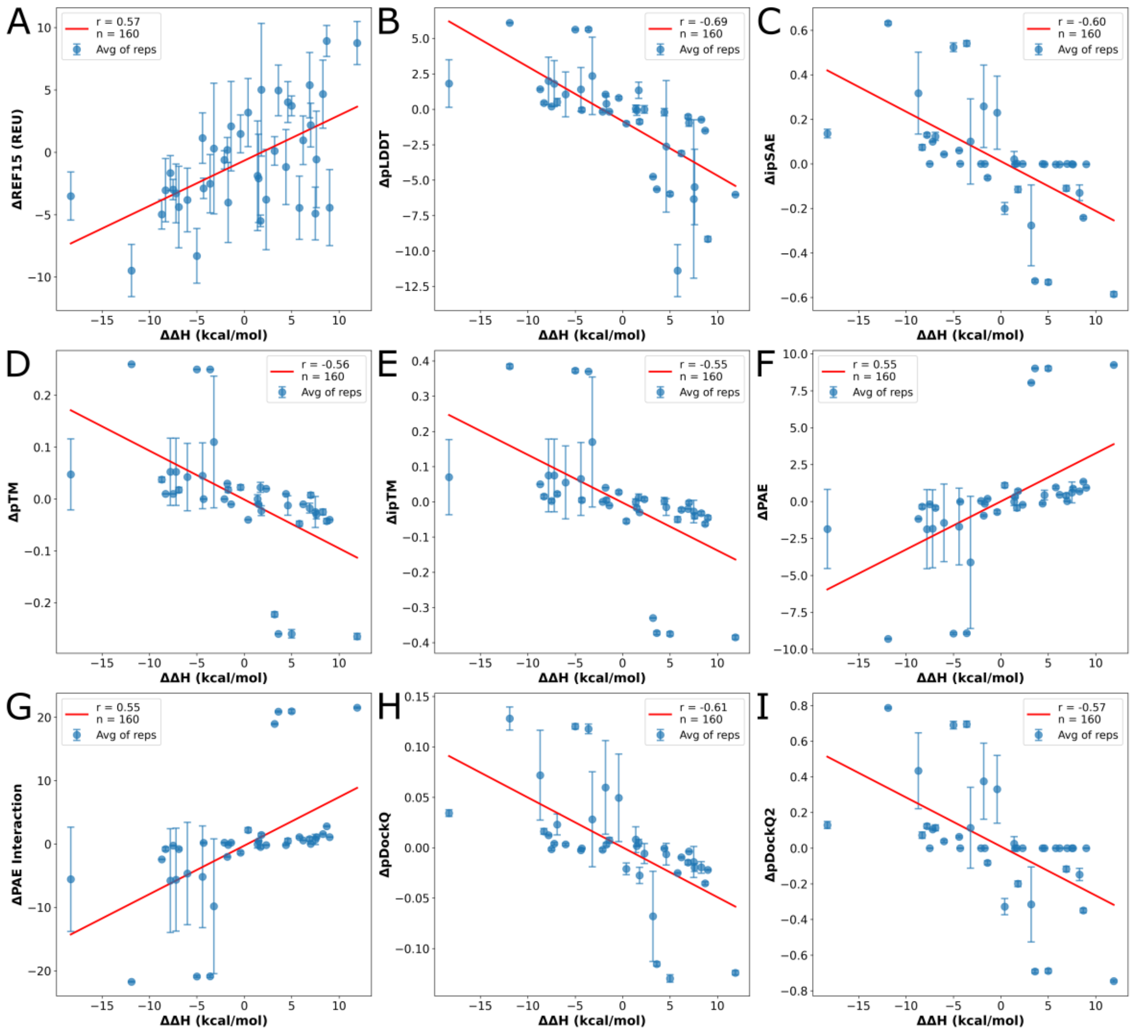

Supplementary Figure 3. Predicted change in metrics versus experimental ΔΔH of SKEMPI Ab-Ag mutations of all types. Blue dots represent the average of all four replicates, with error bars indicating the variation found in the different replicates. The red line represents the linear fit of the predicted and experimentally determined changes in ΔΔH. The represented metrics are (A) REF15; (B) pLDDT; (C) ipSAE; (D) pTM; (E) ipTM; (F) PAE; (G) PAE interaction; (H) pDockQ; (I) pDockQ2.

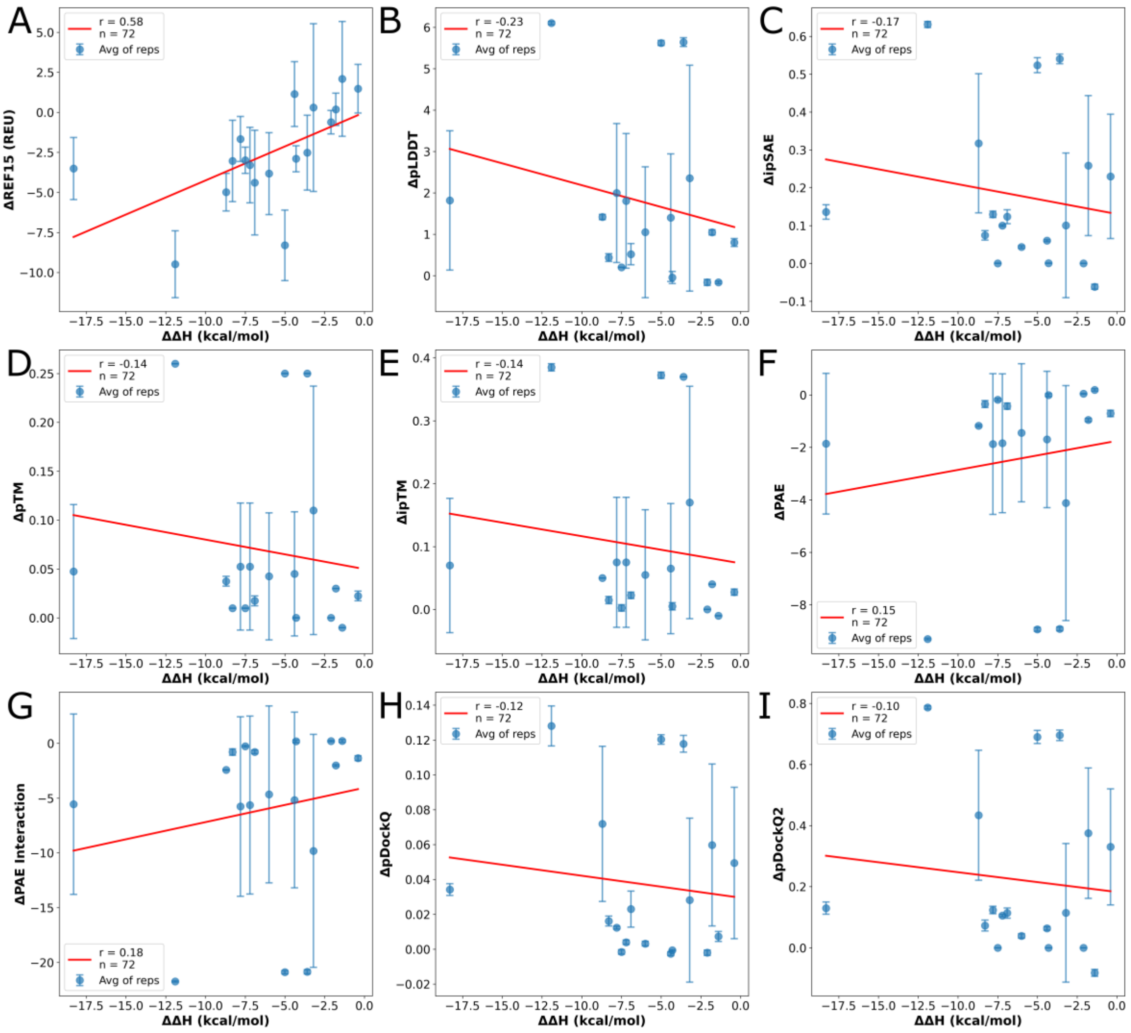

Supplementary Figure 4. Predicted change in metrics versus experimental ΔΔH of SKEMPI Ab-Ag mutations that increase affinity. Blue dots represent the average of all four replicates, with error bars indicating the variation found in the different replicates. The red line represents the linear fit of the predicted and experimentally determined changes in ΔΔH. The represented metrics are (A) REF15; (B) pLDDT; (C) ipSAE; (D) pTM; (E) ipTM; (F) PAE; (G) PAE interaction; (H) pDockQ; (I) pDockQ2.

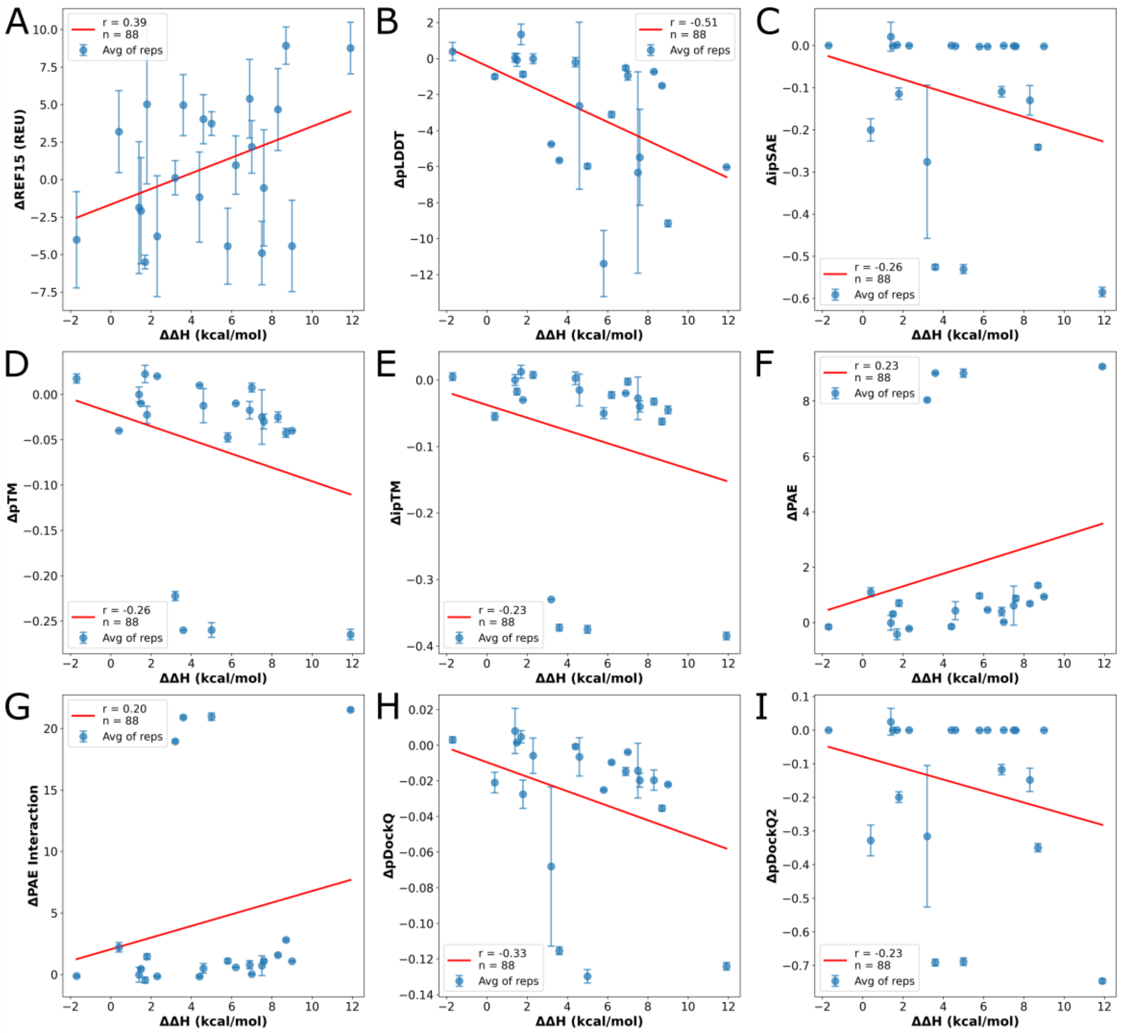

Supplementary Figure 5. Predicted change in metrics versus experimental ΔΔH of SKEMPI Ab-Ag mutations that decrease affinity. Blue dots represent the average of all four replicates, with error bars indicating the variation found in the different replicates. The red line represents the linear fit of the predicted and experimentally determined changes in ΔΔH. The represented metrics are (A) REF15; (B) pLDDT; (C) ipSAE; (D) pTM; (E) ipTM; (F) PAE; (G) PAE interaction; (H) pDockQ; (I) pDockQ2.

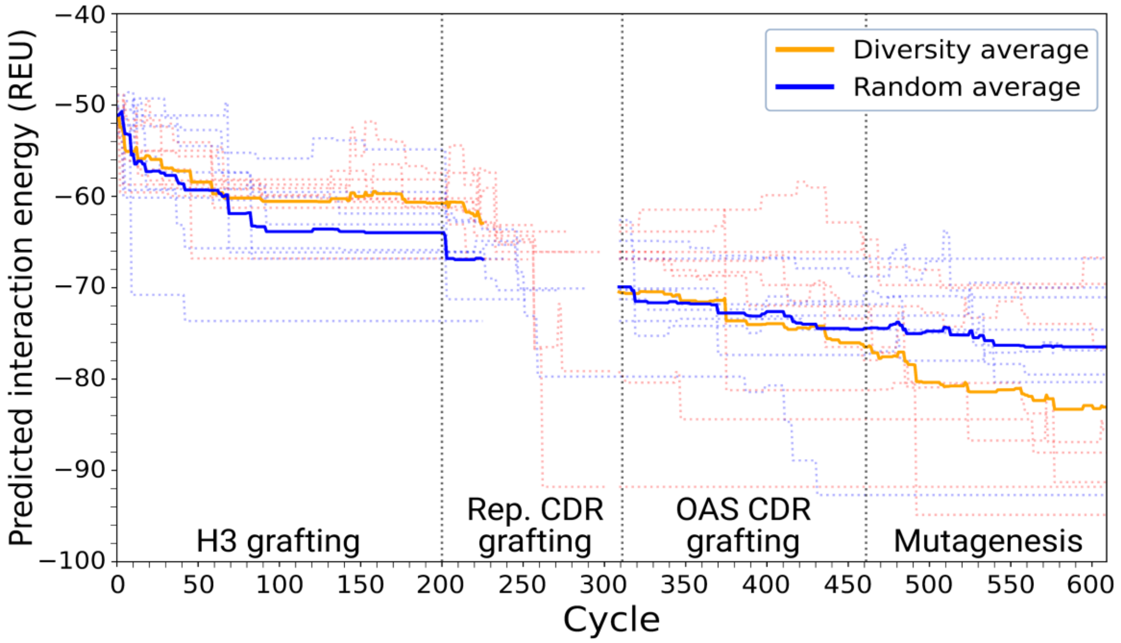

Supplementary Figure 6. Antibody-antigen interaction energy during diversity-guided and randomized optimization of trastuzumab with Ab-SELDON. Average lines were not plotted for cycles where some (but not all) replicates had completed the representative CDR grafting step. The vertical dotted lines separate the cycles of the different steps.

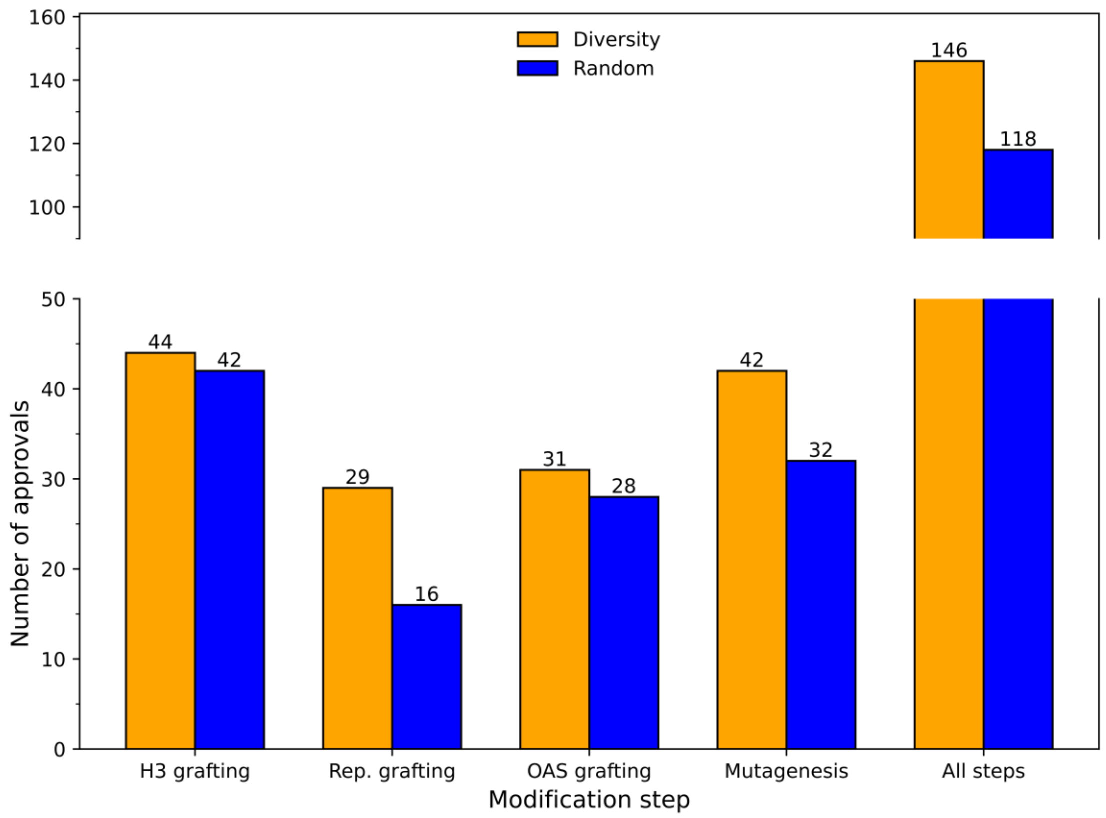

Supplementary Figure 7. Total number of approvals seen for each modification step in diversity-guided (orange) and randomized (blue) optimization runs.

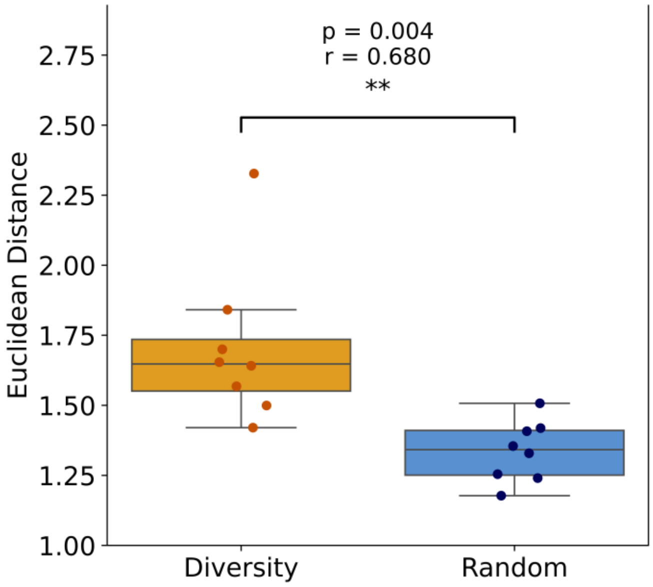

Supplementary Figure 8. Average Euclidean distances between antibody structures inside each group of replicates (diversity and random).

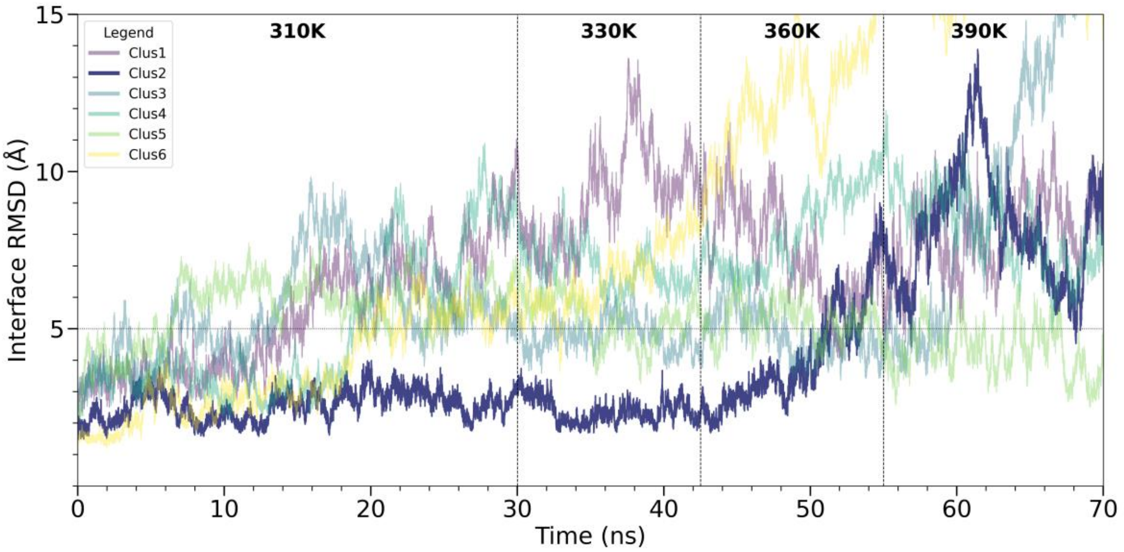

Supplementary Figure 9. iRMSD of the antibody-Gal-3BP complexes produced by docking during hMDs.

Ab-SELDON component	Description
**H3 grafting (H3)**	Module that grafts CDR H3 sequences from naïve OAS antibodies.
**Representative CDR grafting (Rep)**	Module that grafts non-H3 CDR sequences from the PyIgClassify2 canonical structure database.
**OAS CDR grafting (Bnk)**	Module that grafts non-H3 CDR sequences from naïve OAS antibodies.
**Mutagenesis module (Mut)**	Module that tests individual point mutations.
**Framework grafting (Fwk)**	Module that replaces the original framework regions with those from a memory OAS antibody.
**N-CDR**	Dataset with CDR sequences from naïve antibodies; Used by the H3 and OAS CDR grafting modules.
**Me-FWK**	Dataset with framework sequences from memory OAS antibodies; Used by the framework grafting module.
**Mu-FWK**	Subset of Me-FWK with sequences filtered by number of mutations. Used by the framework grafting module.

Supplementary Table 1. Nomenclature of modules and datasets used by the Ab-SELDON pipeline.

Position (Martin)	Structural Context	Top 10% Mutation Rate	Top 20% Mutation Rate	Top 10% Diversity	Top 20% Diversity	Stands out with RA	References
H35	Near H1; VH/VL	YES	YES	YES	YES	YES	[8,14]
H50	Near H2; VH/VL	YES	YES	NO	NO	NO	[12]
H57	Near H2	YES	YES	NO	NO	YES	[12]
H58	Near H2; VH/VL	YES	YES	NO	NO	YES	[12]
H64	Fv/c	NO	YES	NO	NO	NO	N/A
H73	DE Loop	NO	YES	NO	NO	YES	[12,13]
H80	Fv/c	YES	YES	YES	YES	YES	N/A
H81	Fv/c	NO	YES	NO	NO	YES	N/A
H94	Near H3; Vernier zone	YES	YES	YES	YES	YES	[12,13]
L2	Vernier zone	YES	YES	YES	YES	NO	[12,13]
L4	Vernier zone	YES	YES	YES	YES	NO	[12,13]
L6	Hydroph. core	NO	YES	NO	NO	YES	N/A
L9	Fv/c	NO	YES	NO	NO	YES	[54]
L18	Fv/c	NO	YES	NO	NO	YES	N/A
L22	Exposed sheet	NO	YES	NO	NO	YES	N/A

L39	Fv/c	NO	NO	NO	YES	NO	[54]
L43	VH/VL	NO	NO	NO	YES	NO	[9]
L45	VH/VL; Fv/c	YES	YES	YES	YES	YES	[10]
L46	VH/VL; Vernier zone	NO	NO	YES	YES	NO	[11–13]
L49	VH/VL; Vernier zone	YES	YES	NO	YES	YES	[10,12,13]
L76	Fv/c	YES	YES	NO	NO	YES	N/A
L77	Fv/c	YES	YES	NO	NO	YES	N/A
L83	Fv/c	YES	YES	NO	NO	YES	[54]
L85	Fv/c	NO	YES	NO	YES	YES	N/A

Supplementary Table 2. High variability positions in memory antibodies. Columns describe their structural context, their variabilities, whether they stand out using a reduced alphabet (RA) and whether these positions are explicitly mentioned in the literature regarding their importance for the conformation, rigidification, or affinity of the antibody.

CDR/Region	Definition (Martin)	Definition (Rep step)
H1	26-32	23-35
H2	52-56	50-58
H3	95-102	95-102
L1	24-34	24-34
L2	50-56	50-56
L3	89-97	89-97
H4 (DE loop)	71-75	71-75
L4 (DE loop)	66-71	66-71

Supplementary Table 3. CDR and DE loop definitions used by Ab-SELDON. Residue numbers in the Martin scheme. "Rep step" is the non-H3 representative CDR grafting step, which uses definitions based on PyIgClassify2's application of the Aho numbering scheme.

CDR	Naïve	Memory
L1	895692	530524

L2	35848	18314
L3	24182447	2612037
H1	393	37716
H2	953	206164
H3	4.01E+08	14199361

Supplementary Table 4. CDR gamma diversities. Gamma diversities of the different CDRs in both chains of both naïve and memory antibodies.

Metric	N	TP	FN	TN	FP	Precision	Recall	Specificity	F1-Score	Accuracy (%)	Increased Affinity Accuracy (%)	Decreased Affinity Accuracy (%)
**REF15 (Ab-SELDON)**	2204	**325**	**203**	902	774	0.30	**0.62**	0.54	0.40	55.67	**61.55**	53.82
AF3Score Complex CA pLDDT	2204	318	210	1041	635	0.33	0.60	0.62	**0.43**	61.66	60.23	62.11
AF3Score Complex pTM	2204	211	317	1227	449	0.32	0.40	0.73	0.36	65.25	39.96	73.21
AF3Score Complex ipTM	2204	217	311	1220	456	0.32	0.41	0.73	0.36	65.20	41.10	72.79
AF3Score Complex PAE	2204	302	226	993	683	0.31	0.57	0.59	0.40	58.76	57.20	59.25
AF3Score PAE Interaction	2204	305	223	956	720	0.30	0.58	0.57	0.39	57.21	57.77	57.04
ipSAE	2204	320	208	972	704	0.31	0.61	0.58	0.41	58.62	60.61	58.00
pDockQ	2204	256	272	1122	554	0.32	0.48	0.67	0.38	62.52	48.48	66.95
pDockQ2	2204	265	263	1079	597	0.31	0.50	0.64	0.38	60.98	50.19	64.38
Consensus pLDDT + REF15	2204	213	315	**1368**	**308**	**0.41**	0.40	**0.82**	0.41	**71.73**	40.34	**81.62**
Consensus ipSAE + REF15	2204	228	300	1316	360	0.39	0.43	0.79	0.41	70.05	43.18	78.52
Consensus pLDDT + ipSAE	2204	265	263	1207	469	0.36	0.50	0.72	0.42	66.79	50.19	72.02

Supplementary Table 5. Benchmark of scoring functions on all Ab-Ag SKEMPI mutations. Performance of REF15, AF3Score and ipSAE metrics tested on SKEMPI's Ab-Ag mutations. Applies Ab-SELDON's protocol, including modelling with ABodyBuilder2, structural alignment and minimization. TP = True positives; FN = False negatives; TN = True negatives; FP = False positives.

Metric	N	TP	FN	TN	FP	Precision	Recall	Specificity	F1-Score	Accuracy (%)	Increased Affinity Accuracy (%)	Decreased Affinity Accuracy (%)
**REF15 (Ab-SELDON)**	644	**99**	**41**	335	169	0.37	**0.71**	0.66	0.49	67.39	**70.71**	66.47
AF3Score Complex CA pLDDT	644	80	60	383	121	0.40	0.57	0.76	0.47	71.89	57.14	75.99
AF3Score Complex pTM	644	62	78	380	124	0.33	0.44	0.75	0.38	68.63	44.29	75.40
AF3Score Complex ipTM	644	65	75	351	153	0.30	0.46	0.70	0.36	64.60	46.43	69.64
AF3Score Complex PAE	644	75	65	332	172	0.30	0.54	0.66	0.39	63.20	53.57	65.87
AF3Score PAE Interaction	644	76	64	316	188	0.29	0.54	0.63	0.38	60.87	54.29	62.70
ipSAE	644	84	56	306	198	0.30	0.60	0.61	0.40	60.56	60.00	60.71
pDockQ	644	58	82	427	77	0.43	0.41	0.85	0.42	75.31	41.43	84.72
pDockQ2	644	78	62	363	141	0.36	0.56	0.72	0.43	68.48	55.71	72.02

Consensus pLDDT + REF15	644	63	77	**461**	**43**	**0.59**	0.45	**0.91**	**0.51**	**81.37**	45.00	**91.47**
Consensus ipSAE + REF15	644	67	73	437	67	0.50	0.48	0.87	0.49	78.26	47.86	86.71
Consensus pLDDT + ipSAE	644	76	64	395	109	0.41	0.54	0.78	0.47	73.14	54.29	78.37

Supplementary Table 6. Benchmark of scoring functions on multipoint Ab-Ag SKEMPI mutations. Performance of REF15, AF3Score and ipSAE metrics tested on SKEMPI's Ab-Ag multipoint mutations. Applies Ab-SELDON's protocol, including modelling with ABodyBuilder2, structural alignment and minimization. TP = True positives; FN = False negatives; TN = True negatives; FP = False positives.

Threshold (REU)	N	TP	FN	TN	FP	Precision	Recall	Specificity	F1-Score	Accuracy (%)	Increased Affinity Accuracy (%)	Decreased Affinity Accuracy (%)
0.0	644	**99**	**41**	335	169	0.37	**0.71**	0.66	0.49	67.39	**70.71**	66.47
-0.5	644	96	44	347	157	0.38	0.69	0.69	0.49	68.79	68.57	68.85
-1.0	644	91	49	361	143	0.39	0.65	0.72	0.49	70.19	65.00	71.63
-2.0	644	84	56	393	111	0.43	0.60	0.78	**0.50**	74.07	60.00	77.98
-3.0	644	69	71	412	92	0.43	0.49	0.82	0.46	74.69	49.29	81.75
-4.0	644	58	82	**430**	**74**	**0.44**	0.41	**0.85**	0.43	**75.78**	41.43	**85.32**

Supplementary Table 7. Results of tests with multipoint Ab-Ag SKEMPI mutations using Ab-SELDON's protocol. Performance of different minimum predicted energy (REF15) decrease thresholds for approval of mutations, tested with SKEMPI's antibody-antigen mutations. Protocol includes modelling with ABodyBuilder2, structural alignment and minimization. TP = True positives; FN = False negatives; TN = True negatives; FP = False positives. n = 160.

Threshold (REU)	N	TP	FN	TN	FP	Precision	Recall	Specificity	F1-Score	Accuracy (%)	Increased Affinity Accuracy (%)	Decreased Affinity Accuracy (%)
0.0	2204	**233**	**295**	1244	432	**0.35**	**0.44**	0.74	**0.39**	67.01	**44.13**	74.22
-0.5	2204	90	438	1451	225	0.29	0.17	0.87	0.21	69.92	17.05	86.58
-1.0	2204	44	484	1528	148	0.23	0.08	0.91	0.12	71.32	8.33	91.17
-2.0	2204	16	512	1592	84	0.16	0.03	0.95	0.05	72.96	3.03	94.99
-3.0	2204	16	512	1628	48	0.25	0.03	0.97	0.05	74.59	3.03	97.14
-4.0	2204	12	516	**1644**	**32**	0.27	0.02	**0.98**	0.04	**75.14**	2.27	**98.09**

Supplementary Table 8. Results of protocol tests on all Ab-Ag SKEMPI mutations done without modelling or minimization. Performance of different minimum predicted energy (REF15) decrease thresholds for approval of mutations, tested with SKEMPI's antibody-antigen mutations. Mutations were done through PyMOL directly on the

complex, without modelling or minimization. TP = True positives; FN = False negatives; TN = True negatives; FP = False positives.

Threshold (REU)	N	TP	FN	TN	FP	Precision	Recall	Specificity	F1-Score	Accuracy (%)	Increased Affinity Accuracy (%)	Decreased Affinity Accuracy (%)
0.0	2204	**325**	**203**	902	774	**0.30**	**0.62**	0.54	**0.40**	55.67	**61.55**	53.82
-0.5	2204	290	238	959	717	0.29	0.55	0.57	0.38	56.67	54.92	57.22
-1.0	2204	270	258	1009	667	0.29	0.51	0.60	0.37	58.03	51.14	60.20
-2.0	2204	234	294	1123	553	**0.30**	0.44	0.67	0.36	61.57	44.32	67.00
-3.0	2204	172	356	1203	473	0.27	0.33	0.72	0.29	62.39	32.58	71.78
-4.0	2204	138	390	**1295**	**381**	0.27	0.26	**0.77**	0.26	65.02	26.14	**77.27**

Supplementary Table 9. Results of tests with all Ab-Ag SKEMPI mutations using Ab-SELDON's protocol. Performance of different minimum predicted energy (REF15) decrease thresholds for approval of mutations, tested with SKEMPI's antibody-antigen mutations. Protocol includes modelling with ABodyBuilder2, structural alignment and minimization. TP = True positives; FN = False negatives; TN = True negatives; FP = False positives. n = 545.

Threshold (REU)	N	TP	FN	TN	FP	Precision	Recall	Specificity	F1-Score	Accuracy (%)	Increased Affinity Accuracy (%)	Decreased Affinity Accuracy (%)
0.0	644	**41**	**99**	444	60	0.41	**0.29**	0.88	**0.34**	75.31	**29.29**	88.10
-0.5	644	29	111	472	32	**0.48**	0.21	0.94	0.29	**77.80**	20.71	93.65
-1.0	644	4	136	**492**	**12**	0.25	0.03	**0.98**	0.05	77.02	2.86	**97.62**
-2.0	644	4	136	**492**	**12**	0.25	0.03	**0.98**	0.05	77.02	2.86	**97.62**
-3.0	644	4	136	**492**	**12**	0.25	0.03	**0.98**	0.05	77.02	2.86	**97.62**
-4.0	644	4	136	**492**	**12**	0.25	0.03	**0.98**	0.05	77.02	2.86	**97.62**

Supplementary Table 10. Results of tests with multipoint Ab-Ag SKEMPI mutations done without modelling or minimization. Performance of different minimum predicted energy (REF15) decrease thresholds for approval of mutations, tested with SKEMPI's antibody-antigen mutations. Mutations were done through PyMOL directly on the complex, without modelling or minimization. TP = True positives; FN = False negatives; TN = True negatives; FP = False positives. n = 160.

Program/step	Runtime (hh:mm:ss)	Cycles/Gen. Structures	Time per cycle (s)	Settings	Hardware
Ab-SELDON - H3 step	03:34:16	200 cycles	64.3	swap_h3|n_cycles=200	

<table>
<tr><td>Ab-SELDON - Rep step</td><td>02:18:33</td><td>144 cycles</td><td>57.7</td><td>swap_rep|max_cycles=400</td><td rowspan="8">GPU: NVIDIA RTX A6000 48 GB
CPU: Xeon Silver 4216 @ 2.1ghz
RAM: 96GB DDR4</td></tr>
<tr><td>Ab-SELDON - Bnk step</td><td>02:39:40</td><td>150 cycles</td><td>63.9</td><td>swap_bnk|n_cycles=150</td></tr>
<tr><td>Ab-SELDON - Mut step</td><td>02:18:55</td><td>150 cycles</td><td>55.6</td><td>mut|n_cycles=150</td></tr>
<tr><td>Ab-SELDON - Fwk step</td><td>00:42:08</td><td>100 structures
+ 25 cycles</td><td>101.1</td><td>memory_frame|num_hyb_struc=50
memory_frame|num_fullmemory=5</td></tr>
<tr><td>Ab-SELDON (Total)</td><td>11:33:32</td><td>669</td><td>62.2</td><td>-</td></tr>
<tr><td>Ab-SELDON (exc. Fwk)</td><td>10:18:04</td><td>644</td><td>60.7</td><td>-</td></tr>
<tr><td>Rosetta Antibody Design</td><td>15:52:00</td><td>567</td><td>100.7</td><td>-np 32 -outer_cycle_rounds 567</td></tr>
<tr><td>RFantibody</td><td>63:00:00</td><td>567</td><td>400</td><td>-inference.num_designs=567</td></tr>
</table>

Supplementary Table 11. Comparison of runtimes from Ab-SELDON, Rosetta Antibody Design and RFantibody. Computational performance of Ab-SELDON, Rosetta Antibody Design and RFantibody in the optimization of an anti-Gal-3BP antibody. The names of individual Ab-SELDON steps are clarified in Table S1.

REFERENCES

(1) Jost, L. Entropy and Diversity. *Oikos* **2006**, *113* (2), 363–375. https://doi.org/10.1111/j.2006.0030-1299.14714.x.

(2) Lefranc, M.-P.; Giudicelli, V.; Duroux, P.; Jabado-Michaloud, J.; Folch, G.; Aouinti, S.; Carillon, E.; Duvergey, H.; Houles, A.; Paysan-Lafosse, T.; Hadi-Saljoqi, S.; Sasorith, S.; Lefranc, G.; Kossida, S. IMGT®, the International ImMunoGeneTics Information System® 25 Years On. *Nucleic Acids Research* **2015**, *43* (D1), D413–D422. https://doi.org/10.1093/nar/gku1056.

(3) Zong, F.; Long, C.; Hu, W.; Chen, S.; Dai, W.; Xiao, Z.-X.; Cao, Y. Abalign: A Comprehensive Multiple Sequence Alignment Platform for B-Cell Receptor Immune Repertoires. *Nucleic Acids Research* **2023**, *51* (W1), W17–W24. https://doi.org/10.1093/nar/gkad400.

(4) Murphy, L. R.; Wallqvist, A.; Levy, R. M. Simplified Amino Acid Alphabets for Protein Fold Recognition and Implications for Folding. *Protein Eng* **2000**, *13* (3), 149–152. https://doi.org/10.1093/protein/13.3.149.

(5) Peterson, E. L.; Kondev, J.; Theriot, J. A.; Phillips, R. Reduced Amino Acid Alphabets Exhibit an Improved Sensitivity and Selectivity in Fold Assignment. *Bioinformatics* **2009**, *25* (11), 1356–1362. https://doi.org/10.1093/bioinformatics/btp164.

(6) Ye, Y.; Choi, J.-H.; Tang, H. RAPSearch: A Fast Protein Similarity Search Tool for Short Reads. *BMC Bioinformatics* **2011**, *12*, 159. https://doi.org/10.1186/1471-2105-12-159.

(7) Liang, Y.; Yang, S.; Zheng, L.; Wang, H.; Zhou, J.; Huang, S.; Yang, L.; Zuo, Y. Research Progress of Reduced Amino Acid Alphabets in Protein Analysis and Prediction. *Computational and Structural Biotechnology Journal* **2022**, *20*, 3503–3510. https://doi.org/10.1016/j.csbj.2022.07.001.

(8) Wedemayer, G. J.; Patten, P. A.; Wang, L. H.; Schultz, P. G.; Stevens, R. C. Structural Insights into the Evolution of an Antibody Combining Site. *Science* **1997**, *276* (5319), 1665–1669. https://doi.org/10.1126/science.276.5319.1665.

(9) Khalifa, M. B.; Weidenhaupt, M.; Choulier, L.; Chatellier, J.; Rauffer-Bruyère, N.; Altschuh, D.; Vernet, T. Effects on Interaction Kinetics of Mutations at the VH–VL Interface of Fabs Depend on the Structural Context. *Journal of Molecular Recognition* **2000**, *13* (3), 127–139. https://doi.org/10.1002/1099-1352(200005/06)13:3%253C127::AID-JMR495%253E3.0.CO;2-9.

(10) Masuda, K.; Sakamoto, K.; Kojima, M.; Aburatani, T.; Ueda, T.; Ueda, H. The Role of Interface Framework Residues in Determining Antibody VH/VL Interaction Strength and

Antigen-Binding Affinity. *The FEBS Journal* **2006**, *273* (10), 2184–2194. https://doi.org/10.1111/j.1742-4658.2006.05232.x.
(11) Abhinandan, K. R.; Martin, A. C. R. Analysis and Prediction of VH/VL Packing in Antibodies. *Protein Engineering, Design and Selection* **2010**, *23* (9), 689–697. https://doi.org/10.1093/protein/gzq043.
(12) Chiu, M. L.; Goulet, D. R.; Teplyakov, A.; Gilliland, G. L. Antibody Structure and Function: The Basis for Engineering Therapeutics. *Antibodies* **2019**, *8* (4), 55. https://doi.org/10.3390/antib8040055.
(13) Arslan, M.; Karadag, D.; Kalyoncu, S. Conformational Changes in a Vernier Zone Region: Implications for Antibody Dual Specificity. *Proteins: Structure, Function, and Bioinformatics* **2020**, *88* (11), 1447–1457. https://doi.org/10.1002/prot.25964.
(14) Fernández-Quintero, M. L.; Loeffler, J. R.; Bacher, L. M.; Waibl, F.; Seidler, C. A.; Liedl, K. R. Local and Global Rigidification Upon Antibody Affinity Maturation. *Frontiers in Molecular Biosciences* **2020**, *7*.
(15) Petersen, B. M.; Ulmer, S. A.; Rhodes, E. R.; Gutierrez-Gonzalez, M. F.; Dekosky, B. J.; Sprenger, K. G.; Whitehead, T. A. Regulatory Approved Monoclonal Antibodies Contain Framework Mutations Predicted From Human Antibody Repertoires. *Frontiers in Immunology* **2021**, *12*.
(16) Li, T.; Tracka, M. B.; Uddin, S.; Casas-Finet, J.; Jacobs, D. J.; Livesay, D. R. Rigidity Emerges during Antibody Evolution in Three Distinct Antibody Systems: Evidence from QSFR Analysis of Fab Fragments. *PLOS Computational Biology* **2015**, *11* (7), e1004327. https://doi.org/10.1371/journal.pcbi.1004327.
(17) Mishra, A. K.; Mariuzza, R. A. Insights into the Structural Basis of Antibody Affinity Maturation from Next-Generation Sequencing. *Frontiers in Immunology* **2018**, *9*, 117. https://doi.org/10.3389/fimmu.2018.00117.
(18) Abanades, B.; Wong, W. K.; Boyles, F.; Georges, G.; Bujotzek, A.; Deane, C. M. ImmuneBuilder: Deep-Learning Models for Predicting the Structures of Immune Proteins. *Commun Biol* **2023**, *6* (1), 1–8. https://doi.org/10.1038/s42003-023-04927-7.
(19) Schrödinger, LLC. The PyMOL Molecular Graphics System, Version 2.6.
(20) Dolinsky, T. J.; Czodrowski, P.; Li, H.; Nielsen, J. E.; Jensen, J. H.; Klebe, G.; Baker, N. A. PDB2PQR: Expanding and Upgrading Automated Preparation of Biomolecular Structures for Molecular Simulations. *Nucleic Acids Research* **2007**, *35* (suppl_2), W522–W525. https://doi.org/10.1093/nar/gkm276.
(21) Case, D. A.; Thomas E. Cheatham, I.; Darden, T.; Gohlke, H.; Luo, R.; Kenneth M. Merz, J.; Onufriev, A.; Simmerling, C.; Wang, B.; Woods, R. J. The Amber Biomolecular Simulation Programs. *Journal of computational chemistry* **2005**, *26* (16), 1668. https://doi.org/10.1002/jcc.20290.
(22) Case, D. A.; Aktulga, H. M.; Belfon, K.; Cerutti, D. S.; Cisneros, G. A.; Cruzeiro, V. W. D.; Forouzesh, N.; Giese, T. J.; Götz, A. W.; Gohlke, H.; Izadi, S.; Kasavajhala, K.; Kaymak, M. C.; King, E.; Kurtzman, T.; Lee, T.-S.; Li, P.; Liu, J.; Luchko, T.; Luo, R.; Manathunga, M.; Machado, M. R.; Nguyen, H. M.; O'Hearn, K. A.; Onufriev, A. V.; Pan, F.; Pantano, S.; Qi, R.; Rahnamoun, A.; Risheh, A.; Schott-Verdugo, S.; Shajan, A.; Swails, J.; Wang, J.; Wei, H.; Wu, X.; Wu, Y.; Zhang, S.; Zhao, S.; Zhu, Q.; Cheatham, T. E. I.; Roe, D. R.; Roitberg, A.; Simmerling, C.; York, D. M.; Nagan, M. C.; Merz, K. M. Jr. AmberTools. *J. Chem. Inf. Model.* **2023**, *63* (20), 6183–6191. https://doi.org/10.1021/acs.jcim.3c01153.
(23) Hopkins, C. W.; Le Grand, S.; Walker, R. C.; Roitberg, A. E. Long-Time-Step Molecular Dynamics through Hydrogen Mass Repartitioning. *J. Chem. Theory Comput.* **2015**, *11* (4), 1864–1874. https://doi.org/10.1021/ct5010406.
(24) Onufriev, A. V.; Case, D. A. Generalized Born Implicit Solvent Models for Biomolecules. *Annual Review of Biophysics* **2019**, *48* (1), 275–296. https://doi.org/10.1146/annurev-biophys-052118-115325.
(25) Anandakrishnan, R.; Drozdetski, A.; Walker, R. C.; Onufriev, A. V. Speed of Conformational Change: Comparing Explicit and Implicit Solvent Molecular Dynamics

Simulations. *Biophys J* **2015**, *108* (5), 1153–1164. https://doi.org/10.1016/j.bpj.2014.12.047.
(26) Myung, Y.; Pires, D. E. V.; Ascher, D. B. CSM-AB: Graph-Based Antibody–Antigen Binding Affinity Prediction and Docking Scoring Function. *Bioinformatics* **2022**, *38* (4), 1141–1143. https://doi.org/10.1093/bioinformatics/btab762.
(27) Alford, R. F.; Leaver-Fay, A.; Jeliazkov, J. R.; O'Meara, M. J.; DiMaio, F. P.; Park, H.; Shapovalov, M. V.; Renfrew, P. D.; Mulligan, V. K.; Kappel, K.; Labonte, J. W.; Pacella, M. S.; Bonneau, R.; Bradley, P.; Dunbrack, R. L. Jr.; Das, R.; Baker, D.; Kuhlman, B.; Kortemme, T.; Gray, J. J. The Rosetta All-Atom Energy Function for Macromolecular Modeling and Design. *J. Chem. Theory Comput.* **2017**, *13* (6), 3031–3048. https://doi.org/10.1021/acs.jctc.7b00125.
(28) Guest, J. D.; Vreven, T.; Zhou, J.; Moal, I.; Jeliazkov, J. R.; Gray, J. J.; Weng, Z.; Pierce, B. G. An Expanded Benchmark for Antibody-Antigen Docking and Affinity Prediction Reveals Insights into Antibody Recognition Determinants. *Structure* **2021**, *29* (6), 606-621.e5. https://doi.org/10.1016/j.str.2021.01.005.
(29) *List of Rosetta command line options*. https://docs.rosettacommons.org/docs/latest/full-options-list (accessed 2025-07-01).
(30) Jankauskaitė, J.; Jiménez-García, B.; Dapkūnas, J.; Fernández-Recio, J.; Moal, I. H. SKEMPI 2.0: An Updated Benchmark of Changes in Protein–Protein Binding Energy, Kinetics and Thermodynamics upon Mutation. *Bioinformatics* **2019**, *35* (3), 462–469. https://doi.org/10.1093/bioinformatics/bty635.
(31) Liu, Y.; Yu, Q.; Wang, D.; Chen, M. AF3Score: A Score-Only Adaptation of AlphaFold3 for Biomolecular Structure Evaluation. *J. Chem. Inf. Model.* **2025**, *65* (15), 8207–8214. https://doi.org/10.1021/acs.jcim.5c00653.
(32) Dunbrack, R. L. Rēs ipSAE Loquunt: What's Wrong with AlphaFold's ipTM Score and How to Fix It. bioRxiv February 14, 2025, p 2025.02.10.637595. https://doi.org/10.1101/2025.02.10.637595.
(33) Cho, H.-S.; Mason, K.; Ramyar, K. X.; Stanley, A. M.; Gabelli, S. B.; Denney, D. W.; Leahy, D. J. Structure of the Extracellular Region of HER2 Alone and in Complex with the Herceptin Fab. *Nature* **2003**, *421* (6924), 756–760. https://doi.org/10.1038/nature01392.
(34) Weisser, N. E.; Sanches, M.; Escobar-Cabrera, E.; O'Toole, J.; Whalen, E.; Chan, P. W. Y.; Wickman, G.; Abraham, L.; Choi, K.; Harbourne, B.; Samiotakis, A.; Rojas, A. H.; Volkers, G.; Wong, J.; Atkinson, C. E.; Baardsnes, J.; Worrall, L. J.; Browman, D.; Smith, E. E.; Baichoo, P.; Cheng, C. W.; Guedia, J.; Kang, S.; Mukhopadhyay, A.; Newhook, L.; Ohrn, A.; Raghunatha, P.; Zago-Schmitt, M.; Schrag, J. D.; Smith, J.; Zwierzchowski, P.; Scurll, J. M.; Fung, V.; Black, S.; Strynadka, N. C. J.; Gold, M. R.; Presta, L. G.; Ng, G.; Dixit, S. An Anti-HER2 Biparatopic Antibody That Induces Unique HER2 Clustering and Complement-Dependent Cytotoxicity. *Nat Commun* **2023**, *14* (1), 1394. https://doi.org/10.1038/s41467-023-37029-3.
(35) Capone, E.; Iacobelli, S.; Sala, G. Role of Galectin 3 Binding Protein in Cancer Progression: A Potential Novel Therapeutic Target. *J Transl Med* **2021**, *19* (1), 405. https://doi.org/10.1186/s12967-021-03085-w.
(36) Jumper, J.; Evans, R.; Pritzel, A.; Green, T.; Figurnov, M.; Ronneberger, O.; Tunyasuvunakool, K.; Bates, R.; Žídek, A.; Potapenko, A.; Bridgland, A.; Meyer, C.; Kohl, S. A. A.; Ballard, A. J.; Cowie, A.; Romera-Paredes, B.; Nikolov, S.; Jain, R.; Adler, J.; Back, T.; Petersen, S.; Reiman, D.; Clancy, E.; Zielinski, M.; Steinegger, M.; Pacholska, M.; Berghammer, T.; Bodenstein, S.; Silver, D.; Vinyals, O.; Senior, A. W.; Kavukcuoglu, K.; Kohli, P.; Hassabis, D. Highly Accurate Protein Structure Prediction with AlphaFold. *Nature* **2021**, *596* (7873), 583–589. https://doi.org/10.1038/s41586-021-03819-2.
(37) Ruffolo, J. A.; Sulam, J.; Gray, J. J. Antibody Structure Prediction Using Interpretable Deep Learning. *Patterns* **2022**, *3* (2), 100406. https://doi.org/10.1016/j.patter.2021.100406.

(38) Ruffolo, J. A.; Chu, L.-S.; Mahajan, S. P.; Gray, J. J. Fast, Accurate Antibody Structure Prediction from Deep Learning on Massive Set of Natural Antibodies. *Nat Commun* **2023**, *14* (1), 2389. https://doi.org/10.1038/s41467-023-38063-x.
(39) The UniProt Consortium. UniProt: The Universal Protein Knowledgebase in 2025. *Nucleic Acids Res* **2025**, *53* (D1), D609–D617. https://doi.org/10.1093/nar/gkae1010.
(40) Olechnovič, K.; Venclovas, Č. VoroMQA Web Server for Assessing Three-Dimensional Structures of Proteins and Protein Complexes. *Nucleic Acids Res* **2019**, *47* (W1), W437–W442. https://doi.org/10.1093/nar/gkz367.
(41) Ambrosetti, F.; Jiménez-García, B.; Roel-Touris, J.; Bonvin, A. M. J. J. Modeling Antibody-Antigen Complexes by Information-Driven Docking. *Structure* **2020**, *28* (1), 119-129.e2. https://doi.org/10.1016/j.str.2019.10.011.
(42) Brenke, R.; Hall, D. R.; Chuang, G.-Y.; Comeau, S. R.; Bohnuud, T.; Beglov, D.; Schueler-Furman, O.; Vajda, S.; Kozakov, D. Application of Asymmetric Statistical Potentials to Antibody–Protein Docking. *Bioinformatics* **2012**, *28* (20), 2608–2614. https://doi.org/10.1093/bioinformatics/bts493.
(43) Kozakov, D.; Hall, D. R.; Xia, B.; Porter, K. A.; Padhorny, D.; Yueh, C.; Beglov, D.; Vajda, S. The ClusPro Web Server for Protein–Protein Docking. *Nat Protoc* **2017**, *12* (2), 255–278. https://doi.org/10.1038/nprot.2016.169.
(44) Abramson, J.; Adler, J.; Dunger, J.; Evans, R.; Green, T.; Pritzel, A.; Ronneberger, O.; Willmore, L.; Ballard, A. J.; Bambrick, J.; Bodenstein, S. W.; Evans, D. A.; Hung, C.-C.; O'Neill, M.; Reiman, D.; Tunyasuvunakool, K.; Wu, Z.; Žemgulytė, A.; Arvaniti, E.; Beattie, C.; Bertolli, O.; Bridgland, A.; Cherepanov, A.; Congreve, M.; Cowen-Rivers, A. I.; Cowie, A.; Figurnov, M.; Fuchs, F. B.; Gladman, H.; Jain, R.; Khan, Y. A.; Low, C. M. R.; Perlin, K.; Potapenko, A.; Savy, P.; Singh, S.; Stecula, A.; Thillaisundaram, A.; Tong, C.; Yakneen, S.; Zhong, E. D.; Zielinski, M.; Žídek, A.; Bapst, V.; Kohli, P.; Jaderberg, M.; Hassabis, D.; Jumper, J. M. Accurate Structure Prediction of Biomolecular Interactions with AlphaFold 3. *Nature* **2024**, *630* (8016), 493–500. https://doi.org/10.1038/s41586-024-07487-w.
(45) Webb, B.; Sali, A. Comparative Protein Structure Modeling Using MODELLER. *Curr Protoc Bioinformatics* **2016**, *54*, 5.6.1-5.6.37. https://doi.org/10.1002/cpbi.3.
(46) Chaves, B.; Sartori, G. R.; Vasconcelos, D. C. A.; Savino, W.; Caffarena, E. R.; Cotta-De-Almeida, V.; Da Silva, J. H. M. Guidelines to Predict Binding Poses of Antibody-Integrin Complexes. *ACS Omega* **2020**, *5* (27), 16379–16385. https://doi.org/10.1021/acsomega.0c00226.
(47) Radom, F.; Plückthun, A.; Paci, E. Assessment of Ab Initio Models of Protein Complexes by Molecular Dynamics. *PLoS Computational Biology* **2018**, *14* (6), 1–13. https://doi.org/10.1371/journal.pcbi.1006182.
(48) Tian, C.; Kasavajhala, K.; Belfon, K. A. A.; Raguette, L.; Huang, H.; Migues, A. N.; Bickel, J.; Wang, Y.; Pincay, J.; Wu, Q.; Simmerling, C. ff19SB: Amino-Acid-Specific Protein Backbone Parameters Trained against Quantum Mechanics Energy Surfaces in Solution. *J. Chem. Theory Comput.* **2020**, *16* (1), 528–552. https://doi.org/10.1021/acs.jctc.9b00591.
(49) Xiong, Y.; Shabane, P. S.; Onufriev, A. V. Melting Points of OPC and OPC3 Water Models. *ACS Omega* **2020**, *5* (39), 25087–25094. https://doi.org/10.1021/acsomega.0c02638.
(50) Fallon, L.; Belfon, K. A. A.; Raguette, L.; Wang, Y.; Stepanenko, D.; Cuomo, A.; Guerra, J.; Budhan, S.; Varghese, S.; Corbo, C. P.; Rizzo, R. C.; Simmerling, C. Free Energy Landscapes from SARS-CoV-2 Spike Glycoprotein Simulations Suggest That RBD Opening Can Be Modulated via Interactions in an Allosteric Pocket. *J. Am. Chem. Soc.* **2021**, *143* (30), 11349–11360. https://doi.org/10.1021/jacs.1c00556.
(51) *Relaxation of Explicit Water Systems*. https://ambermd.org/tutorials/basic/tutorial13/index.php (accessed 2025-03-10).
(52) Salomon-Ferrer, R.; Götz, A. W.; Poole, D.; Le Grand, S.; Walker, R. C. Routine Microsecond Molecular Dynamics Simulations with AMBER on GPUs. 2. Explicit

Solvent Particle Mesh Ewald. *J Chem Theory Comput* **2013**, *9* (9), 3878–3888. https://doi.org/10.1021/ct400314y.
(53) Roe, D. R.; Cheatham, T. E. I. PTRAJ and CPPTRAJ: Software for Processing and Analysis of Molecular Dynamics Trajectory Data. *J. Chem. Theory Comput.* **2013**, *9* (7), 3084–3095. https://doi.org/10.1021/ct400341p.
(54) Nieba, L.; Honegger, A.; Krebber, C.; Plückthun, A. Disrupting the Hydrophobic Patches at the Antibody Variable/Constant Domain Interface: Improved in Vivo Folding and Physical Characterization of an Engineered scFv Fragment. *Protein Eng* **1997**, *10* (4), 435–444. https://doi.org/10.1093/protein/10.4.435.
